# An alpha-herpesvirus employs host HEXIM1 to promote viral transcription

**DOI:** 10.1128/jvi.01392-23

**Published:** 2024-02-16

**Authors:** Ying Wu, Anyang Sun, Qiqi Yang, Mingshu Wang, Bin Tian, Qiao Yang, Renyong Jia, Shun Chen, Xumin Ou, Juan Huang, Di Sun, Dekang Zhu, Mafeng Liu, Shaqiu Zhang, Xin-Xin Zhao, Yu He, Zhen Wu, Anchun Cheng

**Affiliations:** 1 Engineering Research Center of Southwest Animal Disease Prevention and Control Technology, Ministry of Education of the People’s Republic of China, Chengdu, China; 2 Science & Technology Department of Sichuan Province, International Joint Research Center for Animal Disease Prevention and Control of Sichuan Province, Chengdu, China; 3 Key Laboratory of Animal Disease and Human Health of Sichuan Province, Sichuan Agricultural University, Wenjiang, China; 4 Avian Disease Research Center, College of Veterinary Medicine of Sichuan Agricultural University, Wenjiang, China; University of Toronto, Toronto, Ontario, Canada

**Keywords:** HEXIM1, AnHV-1, alpha-herpesvirus, viral gene transcription, RNA polymerase II, P-TEFb

## Abstract

Although it is widely accepted that herpesviruses utilize host RNA polymerase II (RNAPII) to transcribe viral genes, the mechanism of utilization varies significantly among herpesviruses. With the exception of herpes simplex virus 1 (HSV-1) in alpha-herpesviruses, the mechanism by which RNAPII transcribes viral genes in the remaining alpha-herpesviruses has not been reported. In this study, we investigated the transcriptional mechanism of an avian alpha-herpesvirus, Anatid herpesvirus 1 (AnHV-1). We discovered for the first time that hexamethylene-bis-acetamide-inducing protein 1 (HEXIM1), a major inhibitor of positive elongation factor B (P-TEFb), was significantly upregulated during AnHV-1 infection, and its expression was dynamically regulated throughout the progression of the disease. However, the expression level of HEXIM1 remained stable before and after HSV-1 infection. Excessive HEXIM1 assists AnHV-1 in progeny virus production, gene expression, and RNA polymerase II recruitment by promoting the formation of more inactive P-TEFb and the loss of RNAPII S2 phosphorylation. Conversely, the expression of some host survival-related genes, such as SOX8, CDK1, MYC, and ID2, was suppressed by HEXIM1 overexpression. Further investigation revealed that the C-terminus of the AnHV-1 US1 gene is responsible for the upregulation of HEXIM1 by activating its promoter but not by interacting with P-TEFb, which is the mechanism adopted by its homologs, HSV-1 ICP22. Additionally, the virus proliferation deficiency caused by US1 deletion during the early infection stage could be partially rescued by HEXIM1 overexpression, suggesting that HEXIM1 is responsible for AnHV-1 gaining transcription advantages when competing with cells. Taken together, this study revealed a novel HEXIM1-dependent AnHV-1 transcription mechanism, which has not been previously reported in herpesvirus or even DNA virus studies.

**IMPORTANCE:**

Hexamethylene-bis-acetamide-inducing protein 1 (HEXIM1) has been identified as an inhibitor of positive transcriptional elongation factor b associated with cancer, AIDS, myocardial hypertrophy, and inflammation. Surprisingly, no previous reports have explored the role of HEXIM1 in herpesvirus transcription. This study reveals a mechanism distinct from the currently known herpesvirus utilization of RNA polymerase II, highlighting the dependence on high HEXIM1 expression, which may be a previously unrecognized facet of the host shutoff manifested by many DNA viruses. Moreover, this discovery expands the significance of HEXIM1 in pathogen infection. It raises intriguing questions about whether other herpesviruses employ similar mechanisms to manipulate HEXIM1 and if this molecular target can be exploited to limit productive replication. Thus, this discovery not only contributes to our understanding of herpesvirus infection regulation but also holds implications for broader research on other herpesviruses, even DNA viruses.

## INTRODUCTION

Herpesviruses are reported to cause latent and lytic infections in a wide range of animals and humans (1), and they can be divided into three subfamilies, which are Alpha-, Beta-, and Gamma-herpesvirus (2, 3). The genomes of herpesvirus consist of linear double-stranded DNA and are transcribed in a coordinated temporal cascade in the nucleus of host cells. This way of transcription is well controlled and results in the production of viral immediate early genes, early genes, and late genes (4, 5). Different from RNA viruses (such as influenza virus), which encode their own polymerase (6), the transcription of the herpesvirus genome is dependent on the host RNA polymerase II (RNAPII) due to the lack of a viral RNA polymerase (7–9).

The RNAPII-mediated transcription procedure consists of four steps: initiation, pause, elongation, and termination (10), and each step is precisely regulated and coupled with phosphorylation events on the conserved C-terminal domain (CTD) of the largest RNAPII subunit RPB1 (11, 12). The RNAPII CTD is composed of polypeptide repeats consisting of the seven amino acids Y_1_S_2_P_3_T_4_S_5_P_6_S_7_ (13), and the copy number of the RNAPII CTD polypeptide repeats varies among species (14, 15). During the transcriptional cycle, Y1, T4, and all three serine residues of the RNAPII CTD undergo dynamic phosphorylation and dephosphorylation to recruit factors that regulate transcription, RNA processing, and chromatin modification (13, 16–18). Hexamethylene bis-acetamide inducible protein 1 (HEXIM1) is one of the components of the 7SK snRNP complex (19–21), whose most well-known function is as a specific inhibitor of positive transcriptional elongation factor (P-TEFb, which includes CDK9 and CCNT1) in the regulation of RNAPII-mediated transcriptional elongation (22). The transition of RNAPII from promoter-proximal suspension to productive elongation requires the active forms of P-TEFb to phosphorylate the second serine residue of the RNAPII CTD (23, 24). HEXIM1 binds to 7SK snRNA and the CCNT1 subunit of P-TEFb, sequesters P-TEFb in an inactive state, and blocks the conversion of promoter-proximally paused RNAPII into the elongation-competent state (25). Upon stimulation with certain signaling stimuli, P-TEFb dissociates from the 7SK snRNP complex and is then recruited to the promoter by transcriptional activator proteins, mediators, or BRD4, causing conformational changes in 7SK RNP and the release of HEXIM1 (26, 27). After P-TEFb completes its function, excess free P-TEFb can be captured by 7SK snRNP to form an inhibitory protein complex and realize P-TEFb circulation and activity regulation (28).

As a general transcription elongation factor for most cellular genes, it is not surprising that P-TEFb is also a key player in viral biology. Retroviruses, influenza viruses, and many DNA viruses require P-TEFb activity to hijack host RNAPII transcriptional machinery (29–32). Related studies on herpesviruses have found that viruses have evolved strategies to hijack this key factor via viral proteins and use it as a key participant in RNAPII-mediated viral transcription (31). For example, ICP22 and VP16 proteins of herpes simplex virus 1 (HSV-1) (33, 34), K-cyclin proteins of Kaposi’s sarcoma-associated herpesvirus (KSHV) (35), and EBNA2 of Epstein-Barr virus (EBV) have been found to regulate P-TEFb activity (36). As a key negative regulator of P-TEFb, HEXIM1 plays a critical role in the balance between P-TEFb recruitment and dissociation, which makes it an essential molecule for regulating gene expression. HEXIM1 with abnormal expression or altered autoregulation may fail to perform its function in gene regulation. The HIV-1 Tat protein is widely known for hijacking P-TEFb kinase to activate paused RNAPII via two mechanisms. The best-known mechanism is that the Tat protein competes with HEXIM1 for binding to CCNT1 and 7SK, resulting in the recruitment of P-TEFb to the trans-activating RNA stem-loop formed at the 5′-end of viral nascent pre-mRNAs for further relief of promoter-proximal pausing at the viral promoter (37–39). Recent studies indicate that Tat engages the cytoplasmic ubiquitin ligase UBE2O to ubiquitinate HEXIM1, thereby promoting P-TEFb release from the 7SK snRNP and promoting sequestration of HEXIM1 in the cytoplasm, leading to transcriptional activation of HIV genes (40). However, HEXIM1 has not been reported to act on herpesvirus transcription.

Anatid herpesvirus 1 (AnHV-1) is an alpha-herpesvirus of waterfowl in the Anatidae family. It is also known as duck plague virus or duck enteritis virus (41, 42). Here, we investigated the mechanism of HEXIM1 in AnHV-1 gene transcription, aiming to enrich and refine the potential mechanism by which α-herpesviruses recruit host RNAPII machinery to transcribe viral genomes.

## RESULTS

### AnHV-1 regulates HEXIM1 expression both *in vitro* and *in vivo*

**Fig 1:**
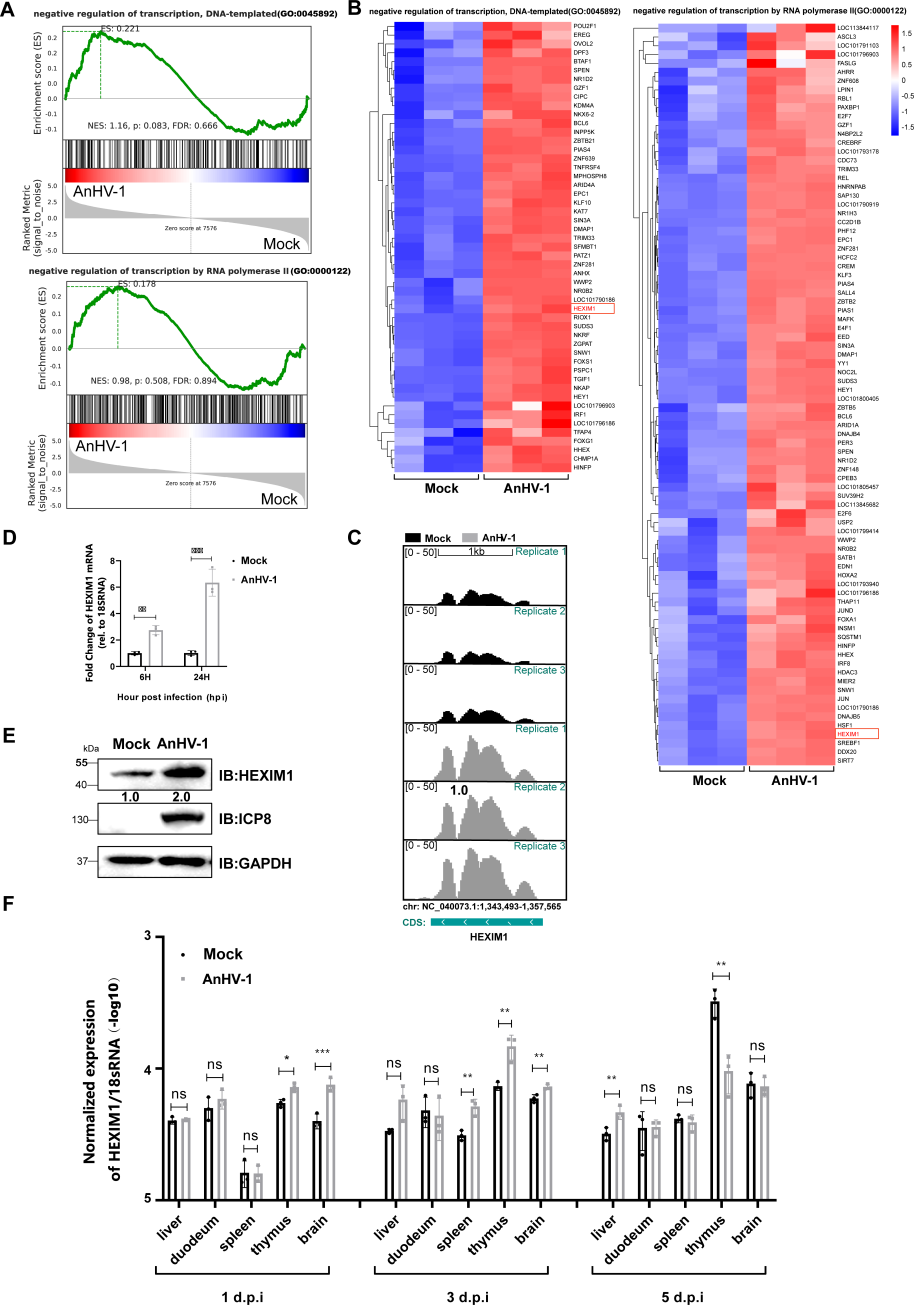
AnHV-1 regulates HEXIM1 expression both *in vitro* and *in vivo*. (**A**) GSEA of gene sets for negative regulation of DNA-templated transcription (top) and negative regulation of transcription by RNA polymerase II (bottom) for AnHV-1- versus mock-infected DEFs. The graph of GSEA analysis corresponded to the result of a gene set analysis: the distribution of enrichment score (ES), the green line was the distribution of ES of all the genes, and the enrichment fraction of the gene set corresponded to the position of the largest absolute value of the *y*-axis. The peak was on the left side of the core gene when ES > 0, and the peak was on the right side of the core gene when ES < 0. NES, normalized enrichment score. FDR, false discovery rate. Positive and negative NESs indicate higher and lower expression after AnHV-1 infection, respectively; the gene distribution map of the gene set, the vertical line indicates the position of the gene in the whole sequence of the gene set; color bar represents the color mapping of the sequence matrix and had a positive value, corresponding to red, the larger the value, the redder the value, and vice versa, corresponding to blue. The closer you got to 0, the closer you got to white; the distribution chart of the sorting matrix, such as the distribution of the difference multiple, signal to noise, and so on. (**B**) Heatmaps showing the comparison of RNA-seq data of genes differentially expressed in mock-infected or AnHV-1-infected DEFs. The color scale bar indicates the significantly differentially expressed genes (DEGs) with *Q* value < 0.05 and |log2FC| > 0.58. (**C**) Corresponding Illumina RNA-Seq coverage plots of DEFs infected with AnHV-1 or mock for 12 h. The *Y* axis denotes the coverage range. Canonical CDS regions are indicated by wide blue boxes. (**D and E**) The relative abundance of HEXIM1 mRNA and protein levels after 5 MOI AnHV-1 infection for 24 h. The normalized gene expression in mock-infected cells was set to 1, and GAPDH was used as the loading control. (**F**) The *in vivo* expression of HEXIM1 after AnHV-1 infection at indicated time points. Three samples per organ were collected in the mock and infection groups for RNA extraction and RT-qPCR. The data in panels D and F were performed on at least three biological replicates per sample; *P < 0.05; **P < 0.01; ***P < 0.001; and ns, not significant.

RNA-seq was used to systemically identify the host transcriptomic changes following AnHV-1 infection of duck embryo fibroblasts (DEFs). Gene expression profiles were analyzed in three independent experiments and quantified with mapped sequencing reads to the duck and AnHV-1 genomes. To identify pathways differentially regulated by AnHV-1 infection in DEFs, we analyzed the bulk RNA-seq data using Gene Set Enrichment Analysis (GSEA). GSEA is a computational method used to determine whether a predefined set of genes shows statistically significant or consistent differences between two biological states, such as phenotypes. It is divided into three steps, namely, calculating enrichment scores, estimating the significance level of enrichment scores, and correcting multiple hypothesis verification. As shown in Fig. 1A, two of the gene sets involved in the negative regulation of transcription were highly upregulated after AnHV-1 infection. A heatmap of genes involved in these two pathways was plotted based on fragments per kilobase million (FPKM, the number of reads per kilobase from a gene per million reads) values, and HEXIM1, a common gene related to negative regulation of transcription mediated by RNAPII or DNA-templated transcription, was highly upregulated after AnHV-1 infection (Fig. 1B; Tables S1 and S2). The subsequent coverage plots derived from RNA-seq data, qPCR, and western blotting analysis confirmed that AnHV-1 infection significantly increased HEXIM1 expression in DEFs (Fig. 1C through E). Moreover, the expression of HEXIM1 *in vivo* was also determined during AnHV-1 infection to better elucidate their correlation. As shown in Fig. 1F, the expression of HEXIM1 in the thymus, brain, and liver was successively upregulated by AnHV-1 during the first 3 days of infection, while the opposite trend was observed in the liver (increased) and thymus (decreased) on day 5 post-infections. But apart from that, no obvious differences existed between AnHV-1 and mock groups at the corresponding time points. Although the changes in HEXIM1 expression *in vivo* were less dramatic than *in vitro*, this discrepancy may be attributed to the AnHV-1 injection dose and the selected organs. However, it should not be overlooked that HEXIM1 expression is spatiotemporally regulated by AnHV-1 *in vivo* and is associated with disease progression, indicating that HEXIM1 may play a role in AnHV-1 pathogenesis beyond its involvement in AnHV-1 transcription.

### AnHV-1 infection enhances the interaction between HEXIM1 and CDK9 in a dose- and time-dependent manner

**Fig 2:**
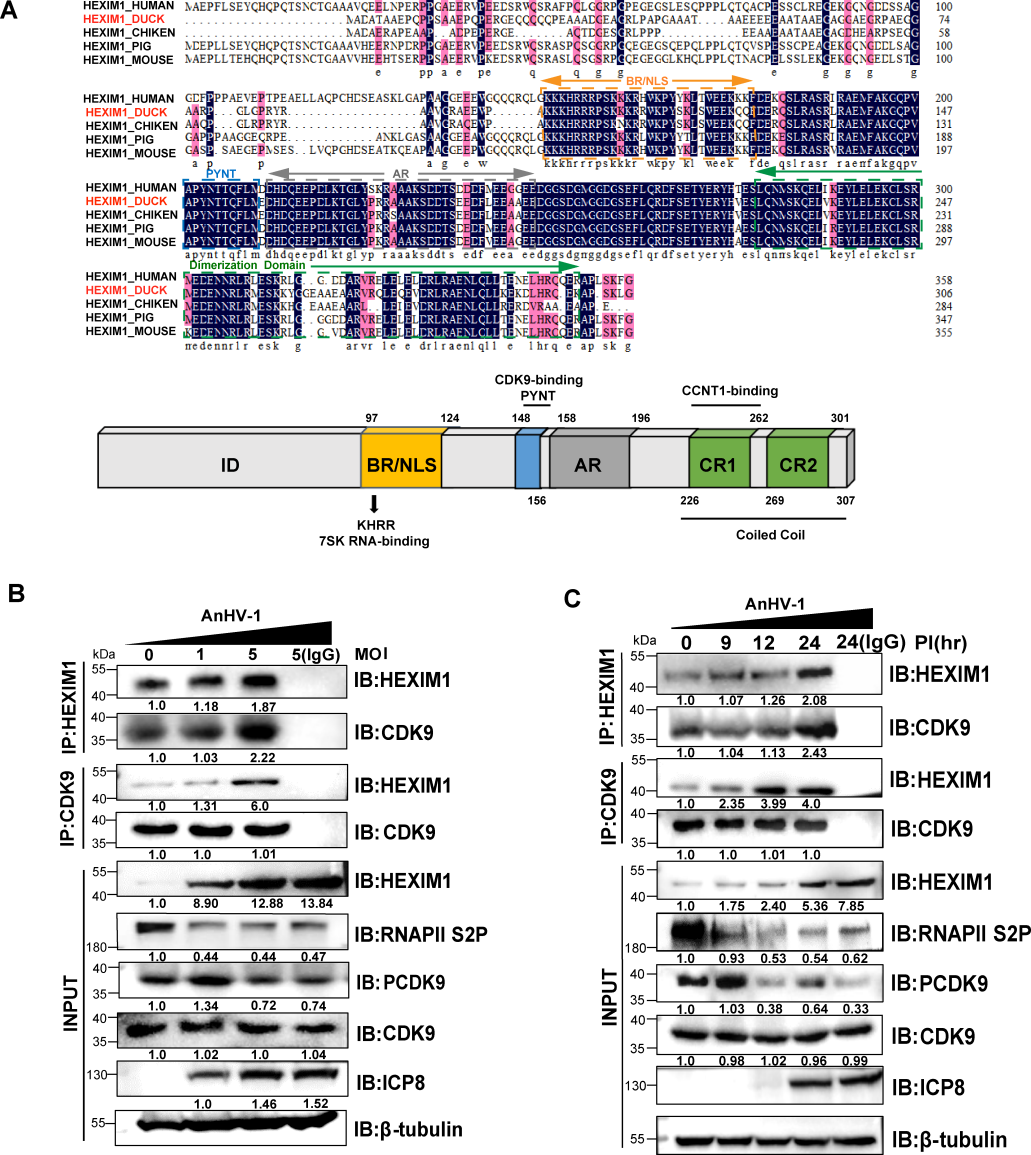
AnHV-1 infection enhances the interaction between HEXIM1 and CDK9 in a dose- and time-dependent manner. (**A**) HEXIM1-related sequences of different species were downloaded from NCBI and analyzed using DNAMAN software for sequence comparison. (**B**) The effect of AnHV-1 infection dose gradients on the expression of HEXIM1 and its interaction with CDK9. DEFs were mock-infected or infected for 24 h with AnHV-1 at an MOI of 1 or 5. (**C**) The effect of AnHV-1 infection time gradients on the expression of HEXIM1 and its interaction with CDK9. DEFs were mock-infected or infected with AnHV-1 at an MOI of 1. The normalized gene expression in mock-infected cells was set to 1, β-tubulin was used as the loading control, and IgG was used as an antibody control in the IP assay.

To further investigate the relationship between HEXIM1 and AnHV-1, we performed sequence alignment of HEXIM1 and found that HEXIM1 was highly conserved across species, especially in the functional domains (Fig. 2A). According to the literature, HEXIM1 N-terminal designated as inhibitory domain was found to inhibit P-TEFb activity and RNA polymerase II transcription (43). BR is a basic region containing a nuclear localization signal (44) and KHRR sequence, which can bind to 7SK snRNA (45). The PYNT region, which was completely conserved among species, was reported to bind P-TEFb when 7SK snRNA bound to the BR region (25, 44), indicating an interaction of HEXIM1 with CDK9 may also occur in ducks. AR is an acidic region of HEXIM1 that interacts with the adjacent BR region in the absence of 7SK snRNA (44), and this interaction can form a self-inhibitory conformation that prevents HEXIM1 from binding to P-TEFb (46). The predicted coiled-coil region in the C-terminal domain of HEXIM1 recognizes the Cyclin T subunit of P-TEFb and forms oligomers in cells (47, 48). We hypothesized that the duck-derived HEXIM1 protein might have functions similar to those of human-derived HEXIM1, which has been the subject of previous studies.

As demonstrated in Fig. 2B and C, we observed a positive correlation between HEXIM1 expression and AnHV-1 infection dose and time, while the levels of CDK9 phosphorylation (pCDK9) and RNAPII Ser2 phosphorylation (RNAPII S2P) showed a negative correlation with the AnHV-1 infection dose and time. The expression of ICP8, a viral load control, confirmed the increased dose and time of AnHV-1 infection. To rule out the possibility that the suppression of pCDK9 was due to the decrease in CDK9 expression after AnHV-1 infection, we also examined the expression of unphosphorylated CDK9. The results showed that AnHV-1 infection did not alter the expression of CDK9 but directly decreased its phosphorylation level. Next, we performed coimmunoprecipitation to detect the effect of AnHV-1 infection on the interaction between CDK9 and HEXIM1. As expected, the high expression of HEXIM1 induced by AnHV-1 promotes the formation of HEXIM1-CDK9 complexes in a virus dose- and infection time-dependent manner (Fig. 2B and C). This may be one of the reasons for the loss of pCDK9 and RNAPII S2P caused by AnHV-1 infection.

### siRNA knockdown of HEXIM1 inhibits AnHV-1 replication and viral gene expression

**Fig 3:**
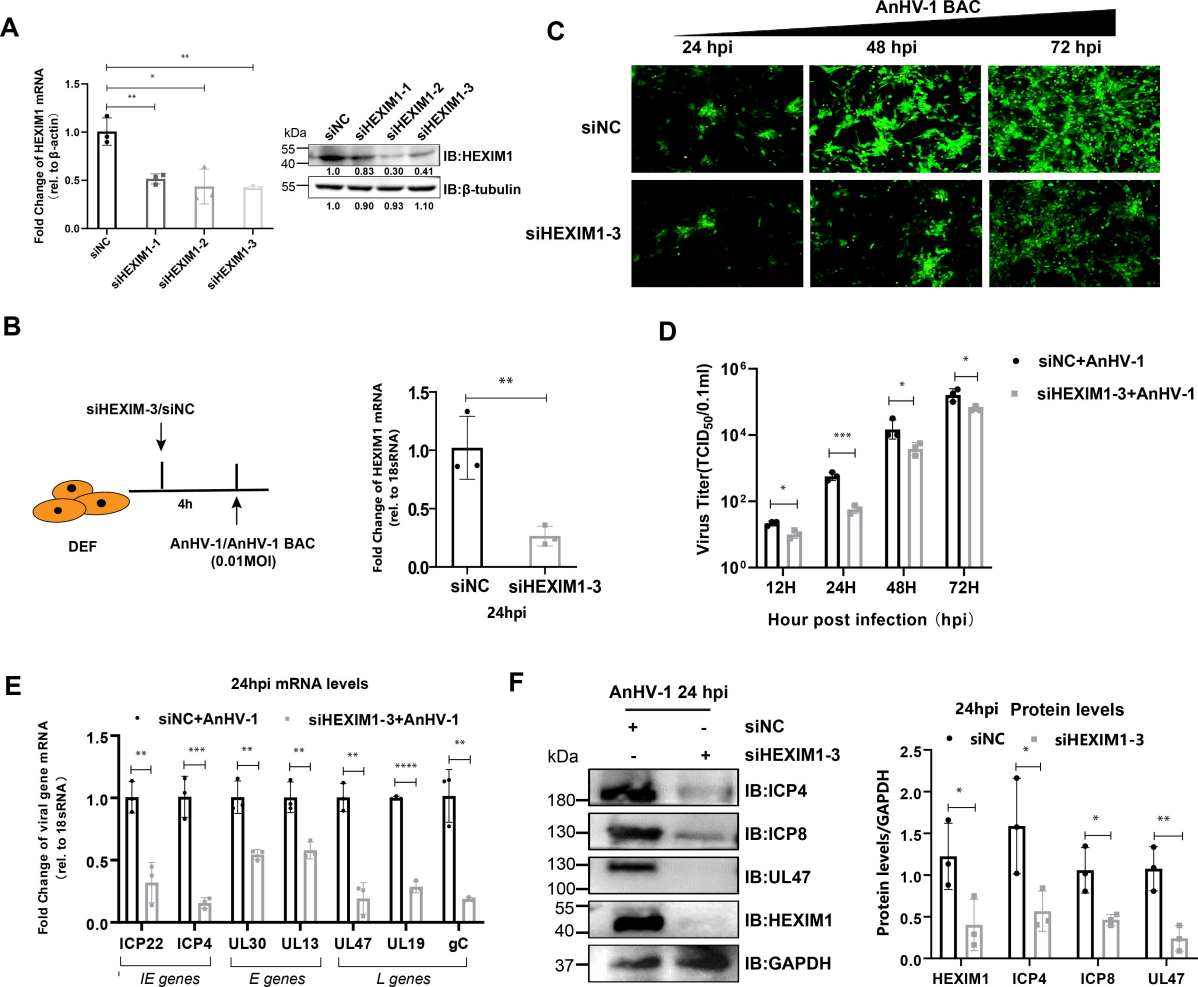
siRNA knockdown of HEXIM1 inhibits AnHV-1 replication and viral gene expression. (**A**) RT-qPCR analysis and western blotting analysis verified the knockdown efficacy of siRNAs targeting the HEXIM1 gene. (**B**) Flow chart for detecting the effect of HEXIM1 knockdown on AnHV-1 replication and gene expression (left) and confirming HEXIM1 knockdown through RT-qPCR (right). (**C–F**) Influence of HEXIM1 knockdown on viral replication (**C**), titer (**D**), gene transcription (**E**), and protein expression (**F**). DEFs transfected with siNC or siHEXIM1-3 were infected with AnHV-1 at an MOI of 0.01. AnHV-1-BAC was a modified virus that had a GFP tag. Image J was used to assess the gray values of three repeated experiments. Data for A, B, D, E, and F were collected using at least three biological replicates per sample. *P < 0.05; **P < 0.01; ***P < 0.001; ****P < 0.0001; and ns, not significant.

In view of previous findings, HEXIM1 expression remained stable during HSV-1 infection (49). This fueled our strong interest in understanding the significance of elevated HEXIM1 expression during AnHV-1 infection. Here, we utilized siRNA to knockdown HEXIM1 and found that all three siRNAs suppressed HEXIM1 transcription and expression (Fig. 3A), and siHEXIM1-3 had better knockdown efficiency, which was selected for the following experiments. A schematic diagram of the HEXIM1 knockdown and AnHV-1 infection assay is shown in Fig. 3B, and qPCR was used to confirm the knockdown efficiency of siHEXIM1-3 after AnHV-1 infection. Then, the effect of siHEXIM1 on AnHV-1 replication was determined. As shown in Fig. 3C, the fluorescent signal representing AnHV-1 BAC infection after siHEXIM1 treatment was significantly less than that in the siNC group at the corresponding detection time point. Knockdown of HEXIM1 resulted in significant downregulation of progeny virus production compared to siNC treatment (Fig. 3D). In addition, the mRNA levels of immediate early genes (ICP22 and ICP4), early genes (UL30 and UL13), and late genes (UL47, UL19, and gC) and the protein levels of ICP4, ICP8, and UL47 were significantly decreased (Fig. 3E and F). To exclude off-target effects of siRNA against HEXIM1, the effect of knockdown HEXIM1 on viral genes’ expression was verified by using multiple siRNAs. As a result, siHEXIM1-1 and siHEXIM1-2 inhibited the transcription and expression of viral genes as we expected (Fig. S1). To confirm whether the suppression of AnHV-1 replication and transcription by siHEXIM1 is connected to the antiviral impact, we employed a dual luciferase reporter assay to investigate the effect of HEXIM1 on activating antiviral gene promoters. As a result, the knockdown of HEXIM1 had no influence on IFNβ promoter activity, suggesting that its effect on AnHV-1 was independent of antiviral response (Fig. S2). At the same time, the IFNβ promoter was inhibited after viral infection for 48 h, as in the previous report (50).

### Overexpression of HEXIM1 facilitates AnHV-1 replication and viral genes’ expression

**Fig 4:**
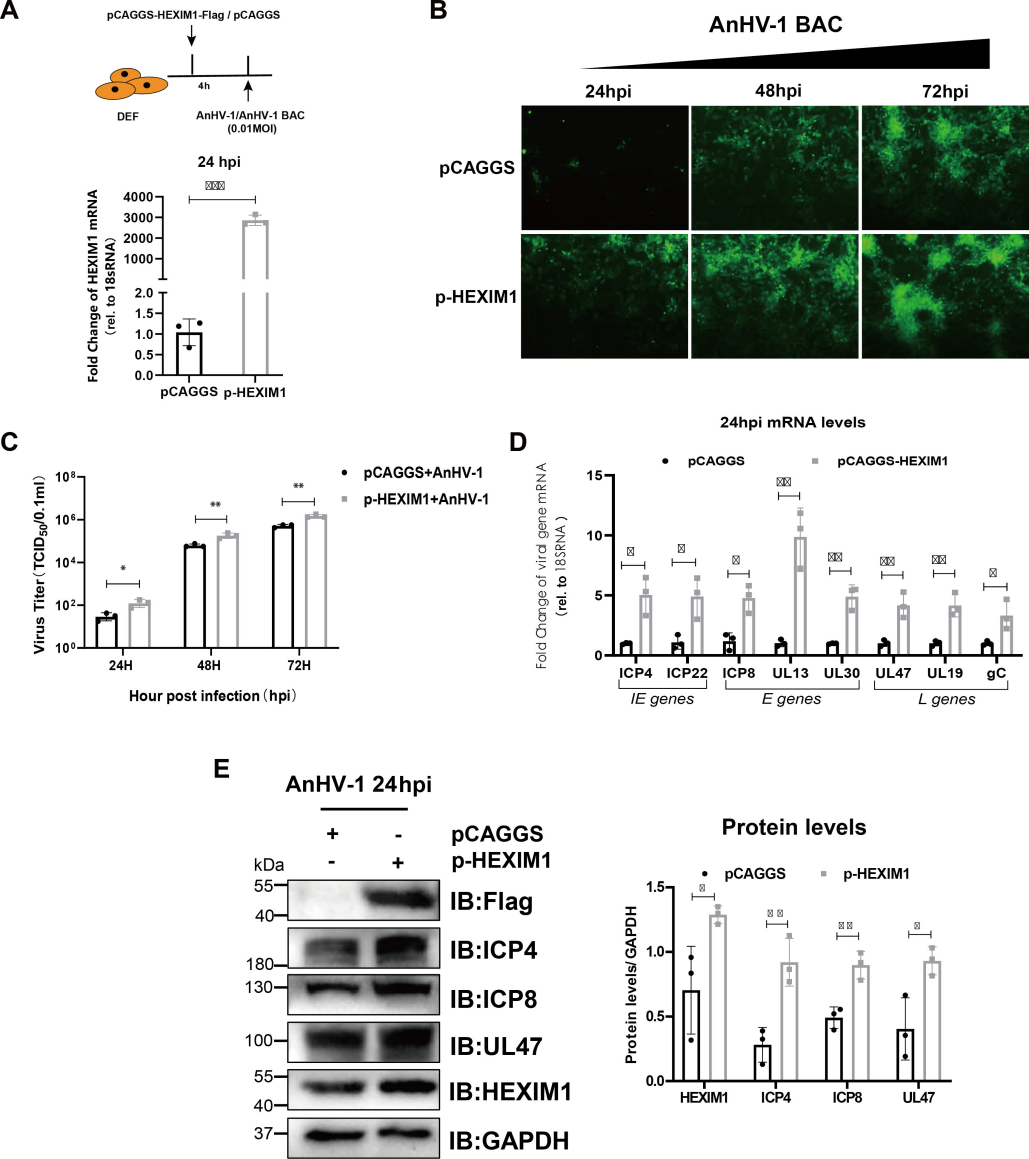
Overexpression of HEXIM1 facilitates AnHV-1 replication and viral gene expression. (**A**) Flow chart for detecting the effect of HEXIM1 overexpression on AnHV-1 replication and gene expression (left), and HEXIM1 overexpression level was assessed by RT-qPCR. (**B–E**) HEXIM1 overexpression promotes viral replication (**B**), titer (**C**), gene transcription (**D**), and protein expression (**E**). DEFs transfected with pCAGGS or pCAGGS-HEXIM1-FLAG eukaryotic plasmids were infected with AnHV-1 at an MOI of 0.01. Image J was used to assess the gray values of three repeated experiments. The data for A, C, D, and E were collected using at least three biological replicates per sample; *P <0.05; **P <0.01; ***P <0.001; and ns, not significant.

To get a more definite conclusion, we next examined the overexpression effect of HEXIM1 on AnHV-1. As shown in Fig. 4A, pCAGGS-HEXIM1-Flag eukaryotic plasmids were transfected to overexpress HEXIM1 ahead of AnHV-1 infection. After the qPCR verification of HEXIM1 overexpression after AnHV-1 infection, we tested the plaque formation of AnHV-1 by using a reporter virus. As shown in Fig. 4B, the infection of AnHV-1 BAC after HEXIM1 overexpression was significantly higher than that in the control group at the same time point. While HEXIM1 overexpression did enhance AnHV-1 proliferation (Fig. 4C), the impact was less pronounced compared to the knockdown of HEXIM1. This could be attributed to the notion that the endogenous HEXIM1 expression in the cells is adequate for efficient virus proliferation at a lower dose (0.01 MOI), as the virus in low-dose infection normally exhibits restricted replication capacity. Then, RT-qPCR and western blotting analysis were performed to examine the mRNA and protein expression levels of viral genes. As shown in Fig. 4D, the mRNA levels of immediate early genes (ICP22 and ICP4), early genes (ICP8, UL30, and UL13), and late genes (UL47, UL19, and gC) were significantly increased. Additionally, the protein levels of the tested viral genes were significantly increased (Fig. 4E). In addition, we repeated the above overexpression experiments by hexamethylene bis-acetamide (HMBA) as an inducer of HEXIM1(66), and its chemical structure is shown in Fig. S3A. To determine the optimal dose and duration for HMBA-induced HEXIM1 transcription and expression, DEFs were treated with HMBA at different doses. As shown in Fig. S3B, 0.5–5 mM HMBA treatment could significantly increase the expression level of HEXIM1 in a dose-dependent manner without affecting cell viability. The dose of 5 mM was selected for subsequent experiments to induce HEXIM1 expression due to its optimal stimulatory result, and we obtained similar results with pCAGGS-HEXIM1-Flag eukaryotic plasmids (Fig. S3C through G). In conclusion, whether HEXIM1 overexpression was induced by transfection of a plasmid or HMBA treatment, we obtained completely opposite results to HEXIM1 knockdown, indicating that HEXIM1 can indeed promote AnHV-1 replication independent of antiviral response.

### HEXIM1 promotes RNAPII S2P recruitment to ICP4 loci

**Fig 5:**
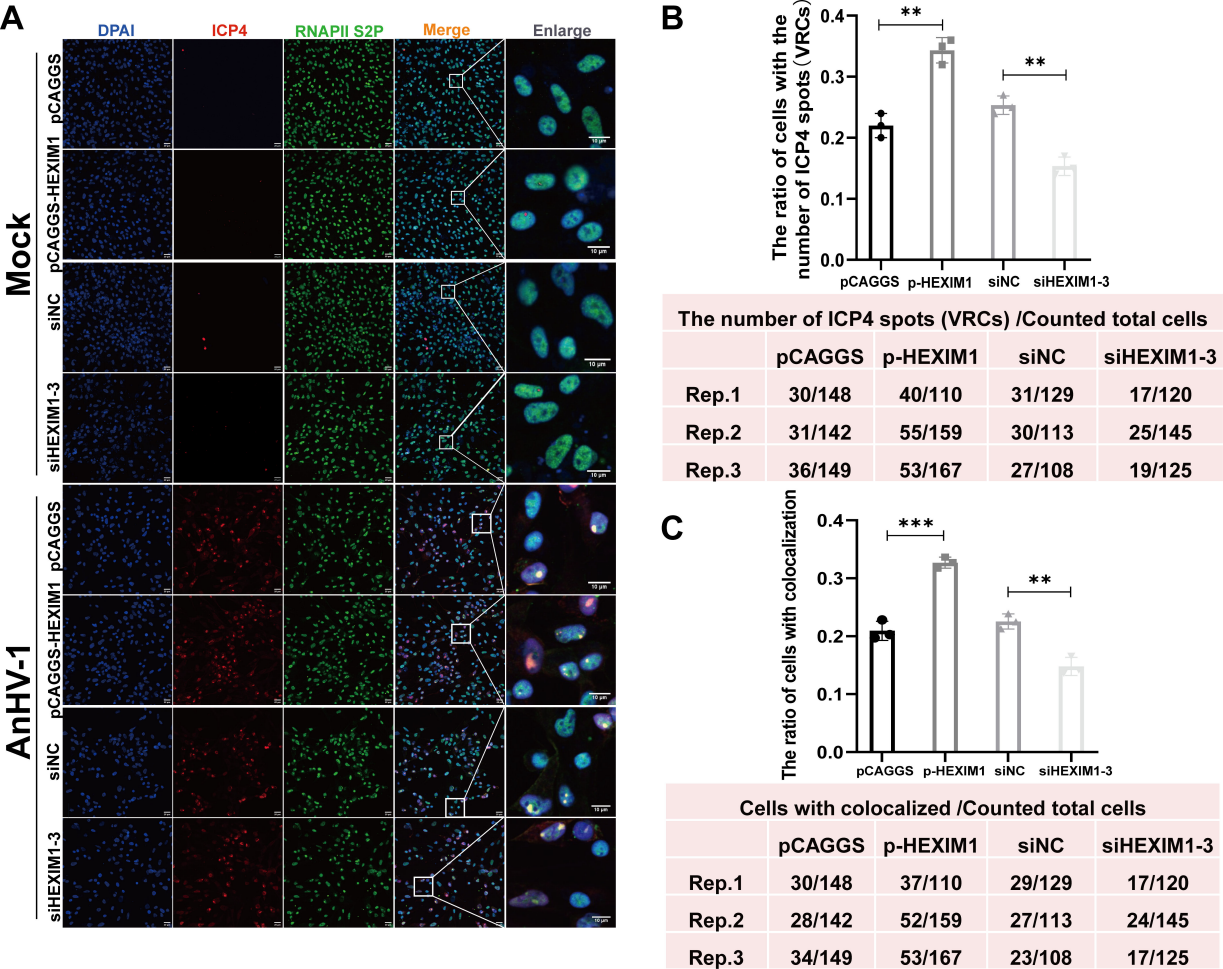
HEXIM1 promotes RNAPII S2P recruitment to ICP4 loci. The co-localization of ICP4 and RNAPII S2P under the overexpression or knockdown of HEXIM1. (**A**) DEFs overexpressing or knockdown of HEXIM1 were mock-infected or infected with AnHV-1 at an MOI of 1. Cells were fixed at 24 hpi and stained with antibodies against ICP4 and RNAPII S2P. Large figures show the typical speckle-like colocalization between ICP4 and RNAPII S2P. (**B**) Bar figure shows the ratio of the number of ICP4 spots (VRCs) among counted total cells. Also shown were the number of cells counted in each repeat. Rep, repeat. (**C**) Bar figure shows the ratio of the number of cells with colocalization between ICP4 and RNAPII S2P among counted total cells. Also shown were the number of cells counted in each repeat. **P < 0.01 and ***P < 0.001.

Viral transcription and DNA replication occur in distinct nuclear domains known as viral replication compartments (VRCs), providing a distinct environment condensed with factors essential for viral transcription and replication (51), as well as allowing the evasion of intrinsic antiviral host responses (52). Previous studies illustrated that VRCs initially identified through the presence of an HSV single-stranded DNA-binding protein, ICP8, originate from small structures that undergo growth, movement, and merging, ultimately occupying the entire nucleus and relocating host chromatin to the nuclear periphery (53, 54). The transcriptional activity of VRCs is confirmed by the presence of the viral transactivator protein ICP4 (55) and RNA polymerase II (56), both of which colocalize with ICP8 (57). Therefore, tracking the formation of VRCs as well as the location of RNAPII during AnHV-1 infection will help in explaining how HEXIM1 influences viral transcription. As shown in Fig. 5A, in the absence of viral infection, RNAPII S2P (the specificity has been tested) was uniformly distributed in the nucleus regardless of whether HEXIM1 was highly expressed or knocked down (upper panel). It aggregates into a punctate distribution in the nucleus and colocalizes with ICP4 loci after AnHV-1 infection, which we defined as VRCs (Fig. 5A, lower panel). Moreover, by statistical analysis of VRC formation and RNAPII S2P recruitment in VRCs after AnHV-1 infection, we found that HEXIM1 promoted VRC formation (Fig. 5B) as well as the colocalization of ICP4 and RNAPII S2P (Fig. 5C), indicating that HEXIM1 contributes to the preferential recruitment of RNAPII S2P to the VRCs by AnHV-1 under the limited host RNAPII S2P.

### HEXIM1 helps AnHV-1 hijack RNAPII but suppresses its binding on host genes

**Fig 6:**
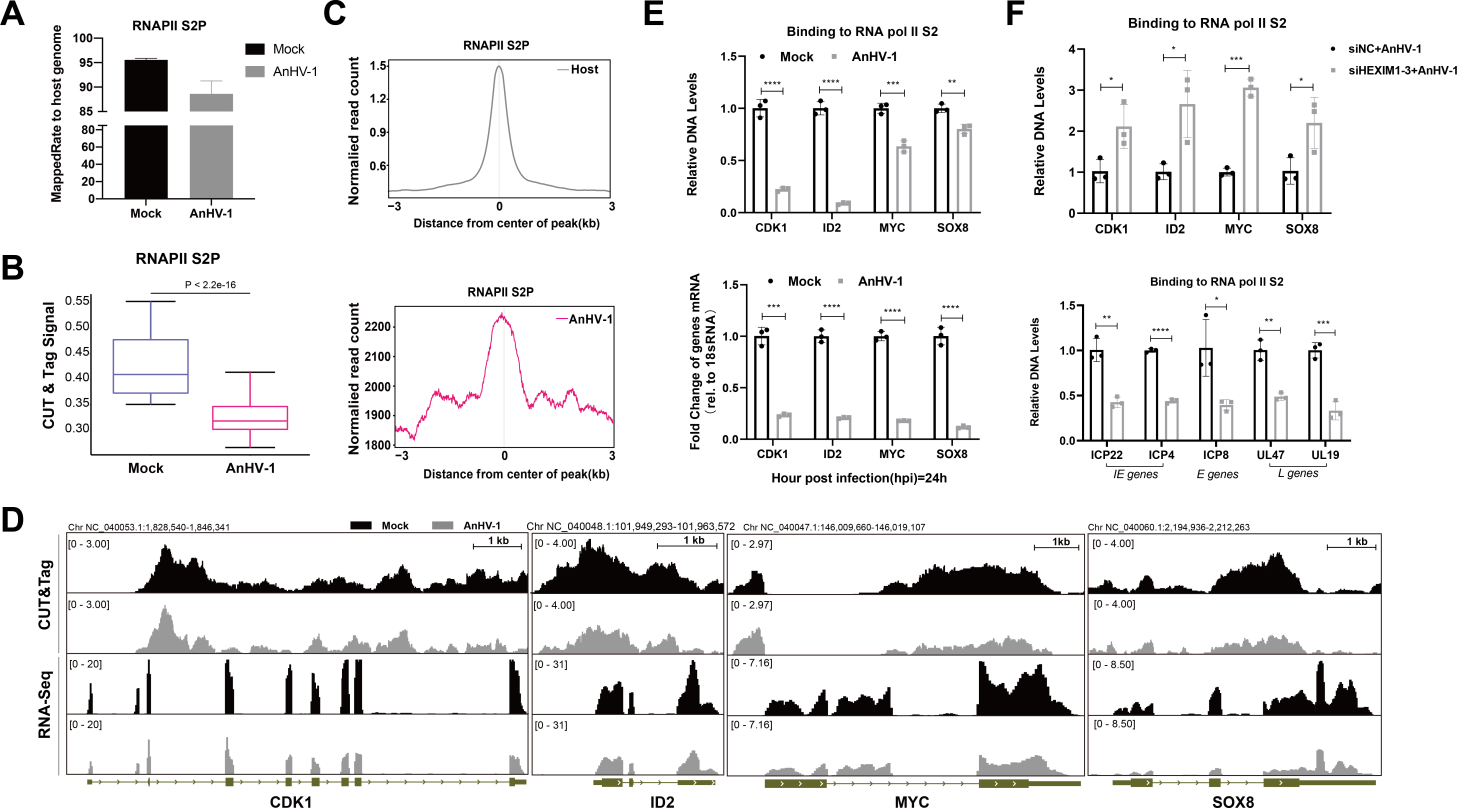
HEXIM1 helps AnHV-1 hijack RNAPII but suppresses its binding on host genes. (**A**) CUT&Tag analysis of the mapping rates of RNAPII S2P to the host genome (the ratio of the sample to the host genome) after infection with AnHV-1 at an MOI of 5 for 6 h, with an RNAPII S2P antibody used for IP. (**B**) Box plot of average RNAPII S2P signals on host genes with/without AnHV-1. (**C**) Average read coverage of RNAPII S2P peaks in the AnHV-1 and host genomes. Read distributions (from BigWig) across peaks are presented as an average plot (average of read signals across all peaks). The horizontal axis represents the normalized peak region coordinates, 0 represents the central point of the peak, negative values indicate how much B is upstream of the peak center, and positive values indicate how much kilobase is downstream of the peak center. The vertical axis represents the average signal value of RNAPII S2P (the normalized read count value). The vertical coordinate was the number of uniform reads aligned to RNAPII S2P. The deepTools tool was used for this analysis. (**D**) Integrated sequencing tracks for RNA-Seq data and RNAPII S2P CUT&Tag data in representative host gene loci. (**E**) RT-qPCR verification of CUT&Tag and RNA-Seq results of representative host genes. (**F**) RNAPII S2P occupancy on representative host and viral genes after AnHV-1 infection with/without HEXIM1 RNA interference. Data in panels I and J were performed with at least three biological replicates per sample; *P < 0.05; **P < 0.01; ***P < 0.001; and ****P < 0.0001.

To better characterize the RNAPII S2P occupancy on viral and host genomes in a global view, we performed RNAPII S2P CUT&Tag experiments with or without AnHV-1 infection. Results showed that RNAPII S2P occupancy across the host genome was significantly decreased after AnHV-1 infection, while the occupancy of RNAPII S2P on the AnHV-1 genome was more than 2,000-fold than that of the host genome (Fig. 6A through C; Table S3). In order to identify the host genes directly regulated by AnHV-1, we conducted combined CUT&Tag and RNA-Seq analyses. Our observations revealed that the transcription of numerous host genes has been inhibited due to the redistribution of RNAPII caused by AnHV-1 infection. This includes cell proliferation-relevant genes, which were among the top 20 items in the GO enrichment analysis of the RNAPII S2P CUT&Tag experiment. As shown in Fig. 6D, there was a marked decrease in RNAPII S2P coverage along representative host genes related to cell growth after AnHV-1 infection, and the mRNA expression of these genes was also decreased in RNA-Seq (Fig. 6D). The subsequent qPCR confirmed the loss of RNAPII S2P occupancy and mRNA levels of CDK1, ID2, MYC, and SOX8 (Fig. 6E). Then, we performed RNAPII S2P CUT&Tag experiments after HEXIM1 knockdown. As expected, the loss of RNAPII S2P occupancy along CDK1, ID2, MYC, and SOX8 under AnHV-1 infection was rescued by HEXIM1 knockdown, while RNAPII S2P enrichment along representative viral genes was significantly impaired (Fig. 6F). Overall, consistent with the conclusion in Fig. 5, we concluded that AnHV-1 can efficiently hijack host RNAPII for transcriptional elongation of viral genes despite a significant reduction in the host RNAPII S2P level after AnHV-1 infection. This mechanism is highly relevant to HEXIM1.

### AnHV-1 US1 promotes HEXIM1 expression independent of interaction with P-TEFb

**Fig 7:**
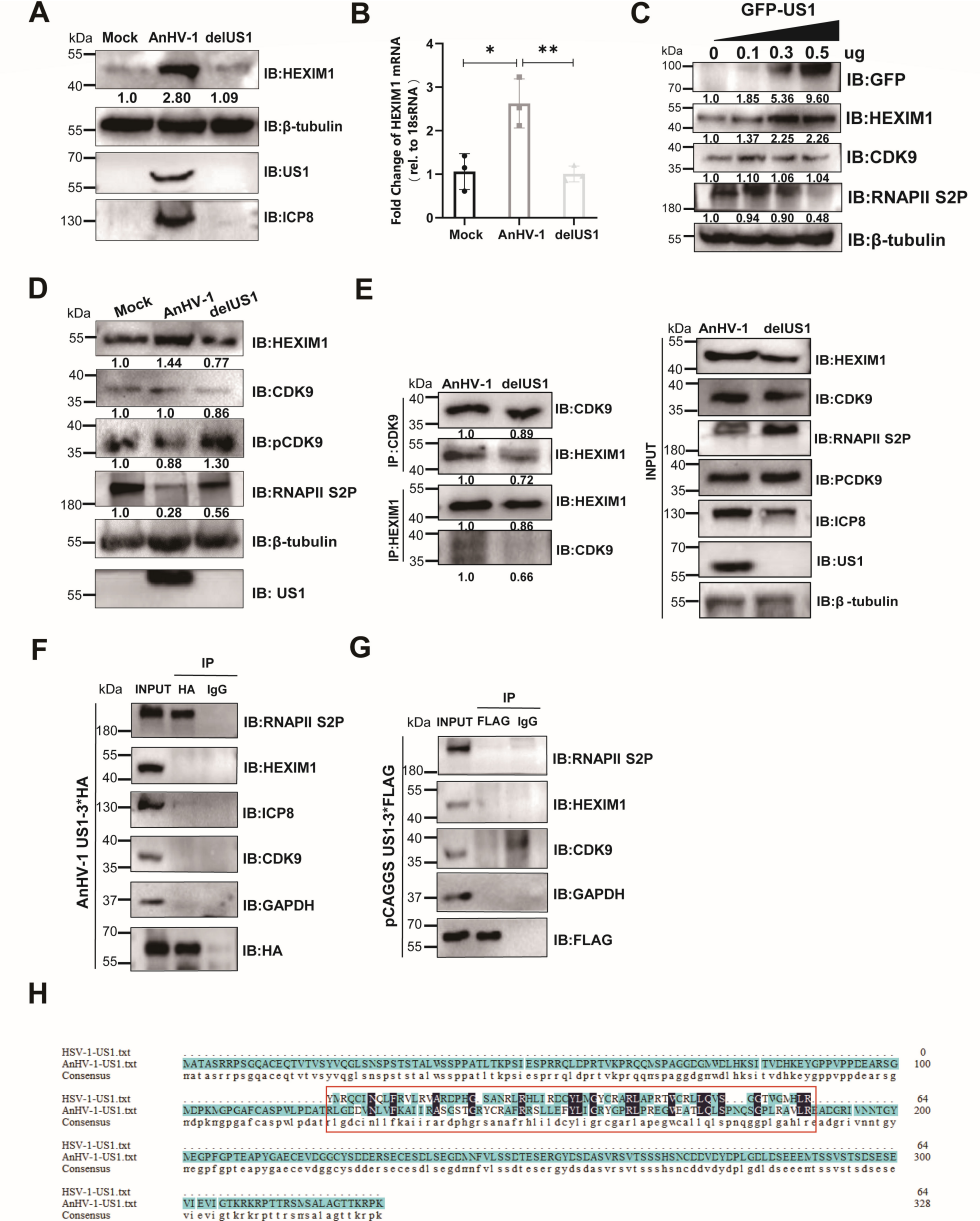
AnHV-1 US1 promotes HEXIM1 expression independent of interaction with P-TEFb. (**A**) DEFs were mock-infected or infected with AnHV-1 or AnHV-1-delUS1 at an MOI of 1. Cell lysates were collected at 24 hpi for western blot analysis. The normalized gene expression in mock-infected cells was set to 1; GAPDH and ICP8 were used as the loading controls. (**B**) DEFs infected with AnHV-1/AnHV-1-delUS1 at an MOI of 1. Total RNA was collected at 24 hpi. The mRNA levels of HEXIM1 were quantified by RT-qPCR. Experiments were performed with at least three biological replicates per sample; *P < 0.05 and **P < 0.01. (**C**) The effect of the US1 plasmid on HEXIM1/CDK9/pCDK9/RNAPII S2P expression. Cell lysates were collected at 24 hpi. The normalized gene expression in the control group was set to 1; β-tubulin was used as the loading control. (**D**) Western blotting analysis of CDK9/pCDK9/RNAPII S2P with/without the US1 gene. Cell lysates were collected at 24 hpi. The normalized gene expression in mock infected cells was set to 1; β-tubulin was used as the loading control. (**E**) The effect of US1 on interaction between HEXIM1 and CDK9. Cell lysates were collected at 24 hpi. Lysates of cells were analyzed via IP with an anti-CDK9 antibody or an anti-HEXIM1 antibody, followed by immunoblotting using host or viral gene antibody. The normalized gene expression in AnHV-1-infected cells was set to 1; β-tubulin was used as the loading control. (**F and G**) The interaction of US1 with CDK9/HEXIM1. DEFs were infected with AnHV-1 US1-3*HA at an MOI of 5 (**F**) or transfected with pCAGGS US1-3*Flag (**G**); cell lysates were collected at 24 hpi. Immunoprecipitation was performed using the corresponding HA/FLAG antibody, followed by immunoblotting using host or viral gene antibody, and IgG was used as the antibody control. (**H**) Sequence alignment between HSV-US1 193-256AA and AnHV-1-US1.

In the aforementioned studies, we clarified that AnHV-1 can preferentially use limited RNAPII S2P by upregulating HEXIM1 expression. This mechanism of hijacking host RNAPII has not been identified in other herpesviruses, which is very attractive to us. To find the specific viral proteins that upregulated HEXIM1 expression, we reviewed the literature and found a homolog of AnHV-1-US1 shown to be involved in the loss of phosphorylation of RNAPII S2P in HSV-1 by directly interacting with CDK9 (33, 58). We used the US1-null mutant to verify whether HEXIM1 expression is upregulated by US1 of AnHV-1. As shown in Fig. 7A and B, a significant reduction of HEXIM1 protein and mRNA expression was observed in the US1 deletion mutant group compared to that of the wild-type group. Moreover, the expression of HEXIM1 could be induced by a GFP-US1 fusion plasmid in a dose-dependent manner, while the phosphorylation level of RNAPII S2 was negatively regulated (Fig. 7C), indicating that the US1 gene was sufficient to induce HEXIM1 expression and thereby influence the pCDK9/RNAPII S2P level (Fig. 7D). More than that, we observed more CDK9 locking in the HEXIM1-CDK9 complex in the presence of US1 due to the high expression of HEXIM1, which could be a reasonable explanation for the loss of pCDK9 and RNAPII S2P in AnHV-1-infected cells (Fig. 7E). However, different from its homologs in HSV-1, there is no interaction between HEXIM1 or CDK9 with AnHV-1 US1, neither by infection with AnHV-1 US1-3*HA nor by transfection with the pCAGGS US1-3*Flag plasmid (Fig. 7F and G), Despite this, another phenomenon worth noting was that US1 and RNAPII S2P could interact with each other at the viral infection level but not at the plasmid transfection level, suggesting that the interaction between US1 and RNAPII S2P requires the participation of other viral proteins. Sequence alignment of the HSV-1-US1 195-256AA domain (33) with the full length AnHV-1 US1 suggested that the known domain interacting with CDK9 in HSV-1 was not conserved among different viruses (Fig. 7H). This may account for the difference in the mechanism by which HSV-1 US1 and AnHV-1 US1 reduce RNAPII S2P phosphorylation levels, which needs to be further elucidated in the future.

### US1 gene directly promotes HEXIM1 promoter activity through its C terminus

**Fig 8:**
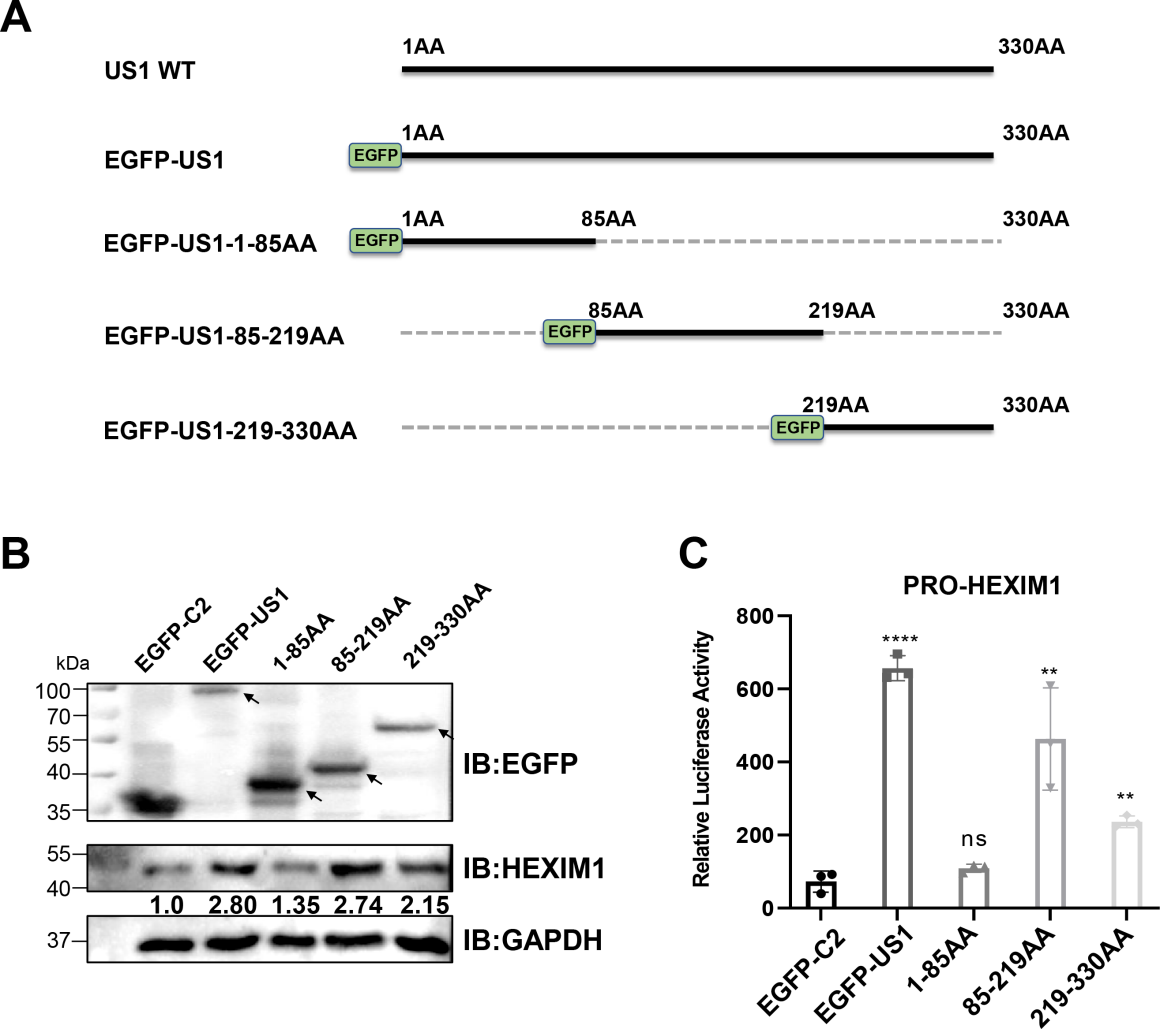
US1 gene directly promotes HEXIM1 promoter activity through its C terminus. (A) Schematic representation of US1 truncated plasmid construction. (B) Western blotting analysis verified the effects of different truncated plasmids of US1 on HEXIM1 expression. Cell lysates were collected at 24 hpi. The normalized gene expression in the EGFP-C2 group was set to 1, and GAPDH was used as the loading control. (C) DEF cells were transfected with pGL-HEXIM1, pRL-TK, and EGFP-C2/EGFP-US1/EGFP-US1-1-85AA/EGFP-US1-85-219AA/EGFP-US1-219-330AA. Cell lysates were collected at 24 hpi. Dual luciferase reporter assay was used to detect the effect of US1 on the activity of the HEXIM1 promoter. The data were performed with at least three biological replicates per sample; **P < 0.01; ****P < 0.0001; and ns, not significant.

In our previous experiments, we found that the US1 gene of AnHV-1 could directly promote HEXIM1 transcription. To investigate which key region of the US1 gene regulates HEXIM1 expression and its level of regulation, we constructed three truncated plasmids (1-85AA, 85-219AA, and 219-330AA) of the US1 gene (Fig. 8A) and detected its promoter activity after co-transfection with HEXIM1 promoter. Western blotting experiments showed that all truncated US1 plasmids were expressed, and the C-terminal region of the US1 gene (85-219AA and 219-330AA) significantly promoted HEXIM1 expression (Fig. 8B). In addition, as expected, the full-length US1 plasmid significantly activated the promoter of HEXIM1, indicating that the US1 gene can promote HEXIM1 mRNA synthesis at the transcriptional level. At the same time, when only the N terminus of the US1 gene was expressed, the US1 gene lost the ability to activate the HEXIM1 promoter, while its C terminus (85-219AA and 219–330AA) had different degrees of activation on HEXIM1 promoter (Fig. 8C). These results indicated that the C terminus (85-330AA) of the US1 gene could directly promote HEXIM1 mRNA synthesis.

## DISCUSSION

P-TEFb, which comprises CDK9 kinase and CCNT1, is a major regulator of RNAPII-driven transcriptional elongation. The transition from active to inactive P-TEFb is dynamic and tightly regulated via HEXIM1 or other factors (19, 28). Extensive literature has demonstrated that the activity of P-TEFb is critical to HSV, HCMV, EBV, HIV, HTLV, HAdV, DENV, and KSHV infection, and several viral proteins have been identified as P-TEFb interaction partners (31). However, as a major inhibitor of P-TEFb, the connection of HEXIM1 to virus infection has been limited to HIV (39). Surprisingly, when using RNA-Seq to analyze the differentially expressed genes after AnHV-1 infection, we found that HEXIM1 was significantly upregulated by AnHV-1, while its expression level remained stable before and after HSV-1 infection (49). This caught our attention, and we realized that although AnHV-1 and HSV-1 belong to the same herpesvirus subfamily alpha, there may be differences in the mechanisms by which they utilize host RNAPII. Therefore, we conducted the present study to explore the role and regulatory mechanism of HEXIM1 in AnHV-1 replication.

When conducting *in vivo* experiments, we encountered challenges in obtaining a consistent trend in HEXIM1 variation at the detection time point. This indicates that HEXIM1 expression is related to the disease infection process and exhibits different regulation patterns in various tissues. Particularly in the spleen and thymus, significant upregulation was observed, which, we speculate, is related to the characteristic of AnHV-1 mainly invading immune organs. As a well-elucidated transcriptional function inhibitor of CDK9(46), HEXIM1 was reported to bind CDK9 to the 7SK snRNP complex through its PYNT peptide (25). This interaction prevents the release of active CDK9 and the phosphorylation of RNAPII S2, consequently leading to transcriptional repression. While the other two members, MePCE and Larp7, act cooperatively to stabilize 7SK and maintain the integrity of 7SK snRNP (59). In accordance with the literature’s findings, we observed significant alterations in the phosphorylation levels of CDK9 and RNAPII S2 with both HEXIM1 knockdown and overexpression (Fig. S4A). Moreover, both endogenous and exogenously expressed HEXIM1 bound more to CDK9 under the overexpression of HEXIM1 (Fig. S4B), supporting the idea that increased HEXIM1 is sufficient to inhibit p-TEFb activity. Regrettably, we failed to verify the other components of the 7SK snRNP complex due to the unavailability of suitable antibodies specific to ducks. Thus, it is possible that other components of the 7SK snRNP complex are also involved. Even so, the importance of HEXIM1 induction during AnHV-1 infection cannot be overlooked, which provides important clues for studies of RNAPII modulation by AnHV-1.

Among the recognized mechanisms of transcriptional elongation regulation in HSV-1 and HSV-2 infection, the virus tends to regulate infection either by interacting with P-TEFb (33) or specific transcription elongation factors (60, 61). This includes inhibiting super elongation complex formation (49) or recruiting transcription elongation factors through BRD4 (62). Except for that, there have been sporadic studies reporting the regulation of transcription by regulating HEXIM1 modification. For example, over-expression of NPM leads to proteasome-mediated degradation of HEXIM1, resulting in the activation of P-TEFb-dependent transcription (63). HIV recruits UBE2O to ubiquitinate HEXIM1 via TAT protein, thereby promoting the dissociation of the inactive form of P-TEFb and efficient viral transcription elongation (40). This indicates that the mechanism of RNAPII transcriptional elongation regulation is diverse. For the first time, we discovered that high expression of HEXIM1 was induced by AnHV-1, an alpha-herpesvirus, leading to a significant promotion of viral production and gene transcription in AnHV-1. This novel discovery intrigued us, as the expression level of HEXIM1 remained stable during HSV-1 infection (49), suggesting a unique mechanism for AnHV-1 in regulating RNAPII activity.

Several proteins of herpesviruses have been reported to trigger the loss of RNAPII Ser2P by interacting with P-TEFb (33, 60, 64, 65). In contrast to known mechanisms adopted by other herpesviruses, we found that AnHV-1 US1 (which encodes ICP22) can upregulate the expression of HEXIM1 and formation of inactive P-TEFb without interacting with either P-TEFb or HEXIM1. One probable explanation is that the US1 gene is poorly conserved among alpha-herpesviruses, particularly in the area where it interacts with P-TEFb, where sequence similarity is quite low. This indicates that the AnHV-1 US1 gene affects HEXIM1 expression and P-TEFb activity in different ways. The experiments that followed demonstrated that the US1 gene controls HEXIM1 mRNA expression at the transcriptional level but not at the post-transcriptional level. Although we did not examine whether HEXIM1 protein decay or modification is also regulated, the novel finding that US1 regulates HEXIM1 mRNA synthesis also provides new insight into the regulatory mechanism of HEXIM1. These results suggest that some transcription factors controlling HEXIM1 expression may be regulated by the US1 gene.

Excessive HEXIM1 triggers the loss of RNAPII S2 and CDK9 phosphorylation by bonding more CDK9, which is in accordance with common sense (22). Exploring in depth how the virus employs HEXIM1 to maintain efficient transcriptional elongation in a state of reduced host RNAPII S2 phosphorylation is really worthwhile. The association between HEXIM1 and viral replication should first be considered for its antiviral role since previous studies reported that HEXIM1 was operated as a tumor suppressor and is implicated in the regulation of innate immunity against KSHV by activating the cGAS-STING-IRF3 signaling pathway (66). However, we found that duHEXIM1 regulated viral replication without eliciting an antiviral response, which differs from KSHV and points to its direct role in transcriptional regulation.

Using CUT&Tag to precisely locate the binding of RNAPII on the viral and host genomes, it was found that the binding of RNAPII S2P on the viral genome is more than 2,000 times that of the host. The RNA-Seq and IF confirmed that this binding is the extended state of RNAPII S2P rather than RNAPII stacking due to transcription pauses. However, the advantage of AnHV-1 utilizing the limited host RNAPII S2P to transcribe viral genes is greatly lost upon HEXIM1 knockdown. Conversely, some host survival-related genes, such as SOX8, CDK8, MYC, and ID2, were reactivated by HEXIM1 knockdown. Also, the virus proliferation deficiency caused by US1 deletion during the early infection stage could be partially rescued by HEXIM1 overexpression (Fig. S5), suggesting that HEXIM1 is responsible for the transcription advantages of AnHV-1 when competing with cells. Although the current data are not sufficient enough to reveal the specific mechanism by which this occurs, we can see some clues from previous research. Several studies have demonstrated that these seemingly completely conflicting events have been shown to be important viral survival strategies in the studies of other herpesviruses. In earlier studies, a mechanism widely accepted by researchers in the field is that the HSV-1 VP16 protein competes with ICP22 to recruit elongation factors to viral loci, thereby overcoming the viral transcription inhibition caused by ICP22 (34). Additionally, ICP22 itself is involved in recruiting elongation factors like the FACT complex to the HSV-1 genome for efficient viral transcription elongation late in viral infection and infectious virion production (61). A most recent study of HSV-1 ICP22 in transcription based on PRO-Seq and GRO-Seq revealed its negative regulation of transcriptional elongation on viral genes to limit antisense and intergenic transcription on the highly compact viral genome. This regulatory function directly or indirectly helps to retain RNAPII activity on the viral genome later in infection, which helps explain the apparent paradox between RNAPII S2 phosphorylation inhibition and effective viral transcription (67). These findings consistently affirm the positive role of ICP22 in inhibiting RNAPII S2 phosphorylation for efficient HSV-1 replication and reveal a significant advantage in negatively regulating transcriptional elongation. It is also shown that herpesviruses overcome this elongation barrier using more than one strategy. Obviously, AnHV-1 adopted a mechanism that differs from currently known pathways to inhibit RNAPII S2 phosphorylation, but whether AnHV-1 overcomes the transcriptional barrier resulting from ICP22-induced HEXIM1 overexpression through a known or unrevealed way is currently under investigation. It will take some time before these mechanisms are fully revealed, and they will be the focus of our future work.

In summary, the data in this study support the hypothesis that upregulation of HEXIM1 mRNA synthesis by AnHV-1 US1 C-terminus downregulates RNA polymerase elongation on host genes while enhancing or, at least, protecting elongation on viral genes. As such, this may be a previously unrecognized facet of the host shutoff manifested by many DNA viruses. The study raises a number of obvious and interesting mechanistic questions. It will also be interesting to know whether other herpesviruses manipulate HEXIM1 in a similar way and whether this molecular target can be exploited to limit productive replication.

## MATERIALS AND METHODS

### Cell culture and transfection

To prepare duck embryo fibroblasts, 9-day-old healthy duck embryos were purchased from the Chengdu Klimo breeding company. DEFs were propagated in Dulbecco's modified Eagle medium (DMEM, Gibco) supplemented with 10% (vol/vol) newborn calf serum (NBCS, Gibco), 100 units/mL penicillin, and 100 µg/mL streptomycin (Sangon Biotech, E607011). Cells were cultured in a thermostatic cell incubator at 37°C in a humidified atmosphere containing 5% CO_2_ (Thermo Fisher Scientific). The cells were transfected using liposome transfection reagent (YESEN, 40802ES03) and 50 nM nontargeting or HEXIM1-targeting siRNA (Genebiogist) in DMEM (Gibco). Four hours later, the siRNA-containing medium was removed and replaced with antibiotic-free 2% NBS/DMEM; at the same time, the cells were infected with AnHV-1 and then collected at the indicated time points. The cells were transfected with plasmids in the same manner.

### Viruses

AnHV-1-CHv (GenBank accession no. JQ647509.1, Search: Anatid herpesvirus 1 strain CHv, complete genome - Nucleotide - NCBI (nih.gov), AnHV-1-BAC (68), AnHV-1-delUS1, and AnHV-1-US1-3*HA are preserved by the Avian Disease Research Center of Sichuan Agricultural University. According to the experimental requirements, DEF cells were cultured in different cell dishes and infected with viruses at different MOIs. According to the experiment, samples were collected using the corresponding method for the following steps.

### siRNA and plasmid

The following siRNA targeting sequences were designed and obtained from Genebiogist: GACCUGCACAGACAG-GAGAAATT (siHEXIM1-1), CGGCCGCCAAGUCGGA-CGACATT (siHEXIM1-2), and CCCGCAUGGAGGACGAGAATT (siHEXIM1-3). We used the pCAGGS and EGFP-C2 vector to construct the pCAGGS-HEXIM1-Flag/pCAGGS-US1-3*Flag and EGFP-US1/EGFP-I-85AA/EGFP-85-219AA/EGFP-219-330AA plasmid. Primer sequences are shown in Table 1.

**TABLE 1:** Sequences of all primers used in this study

Primer^*a*^	Sequence (5′–3′)
pCAGGS-HEXIM1-Flag-F	CATCATTTTGGCAAAGAATTC GCCACCATGGATTACAAGGATGACGA
pCAGGS-HEXIM1-Flag-R	CGATAAGATGGCAGACGCAACGGCC TTGGCAGAGGGAAAAAGAT
pCAGGS-US1-3*HA-F	CTTGTTACATCCCACCACTG TGTCTCATCATTTTGGCAAAGAATTCATGGATTACAA
pCAGGS- US1-3*HA-R	GGATGACGACGATAAGACTGCGATAGACATGGCGAC GAGGGAAAAAGATCTGCTAGCTC
EGFP-US1-1-85 F	CTACCGGACTCAGATCTCGAGGCCACC ATGGCGACGGCATCGCGA
EGFP-US1-1-85 R	GTACCGTCGACTGCAGAATTCCGCCACC GTGATCTACAGTGATGGAC
EGFP-US1-85-219 F	CTACCGGACTCAGAT CTCGAGGCCACC ATGCACAAGGAATACGGACCT
EGFP-US1-85-219 R	GTACCGTCGACTGCAGAATTCCGCCACC GTCGACCTCACATTCTGCTCC
EGFP-US1-219-330 F	CTACCGGACTCAGAT CTCGAGGCCACC ATGGACGGGGGATGTTATTCTG
EGFP-US1-219-330 R	GTACCGTCGACTGCAGAATTCCGCCACC ACTCTTGGGGCGTTTTGTG
PGL4.10-HEXIM1-F	CTGGCCTAACTGGCC GGTACC AGCGTTGCCTTCTCAGCC
PGL4.10-HEXIM1-R	CAGTACCGGATTGCC AAGCTT CCGTTGCGTCTGCCATGG
HEXIM1 qF	GAGCGCATCAGAGGCACTTTCCG
HEXIM1 qR	GCCAAGTCGGACGACACGA
18sRNA qF	GCAGGCTCTCCACATGGTA
18sRNA qR	GTACAGTGAAACTGCGAATGG
ICP22 qF	CGTCGGCATGTATTAGCTCTA
ICP22 qF	CGTAGCGTCACATCAAGCAG
ICP4 qF	GCGTTTGGTCCCTATAACCTC
ICP4 qR	AATCTATGCCCGTCCAAGCTC
ICP8 qF	CCCGGACCCATTACTAGGCACA
ICP8 qR	GGCAATACATGAAGATGGAG
UL30 qF	ATACAATGCAGAGTTGGTAC
UL30 qR	ACTCTCGGGTAATCATACTGG
UL13 qF	TACCGATTCCGTGTTTGTTCG
UL13 qR	CTCTATAAATCCCTCGTTGCA
UL47 qF	TAACTGAACCTCTCCGTAGCC
UL47 qR	AACGGAGTTGCTTGGAGAACA
UL19 qF	TGGGCGATGAAACAGAGTAGG
UL19 qR	ACTTGATTTCCTACGAGCAAC
gC qF	ATATAATCAAGCCAGCGGTCA
gC qR	CGAATCATAAAGGGCCGCATC
CDK1 qF	AGTTGCCAGCTCAGCAAATATG
CDK1 qR	CAGGTCTCCAGAGGTATTGCT
ID2 qF	AGATCGCCTTGGACTCGCACC
ID2 qR	ATGTCTGTGTTGAGGGTGGTCAG
MYC qF	ATCCATCAGCACAACTACGC
MYC qR	GAGCATTTTCGGTTATTACTGA
SOX8 qF	ATCCTACTCCCACTCGGCCACG
SOX8 qR	TGGGACTGGTCACTGTAGTG

^*a*^ qF, qPCR forward; qR, qPCR reverse.

### Dual luciferase reporter assay

DEFs knocked down of HEXIM1 were transfected with pGL-IFNβ, pRL-TK, and pCAGGS-STING-MYC/pCAGGS to testify the effect of HEXIM1 on antiviral response; DEFs were transfected with pRL-TK, pGL-HEXIM1, EGFP-C2/EGFP-US1/EGFP-US1-1-85AA/EGFP-US1-85-219AA/EGFP-US1-219-330AA to testify the effect of US1 on HEXIM1 promoter activity. After 24 h, cells were treated according to the TransDetect Double-Luciferase Reporter Assay Kit (Transgen, TR202-02), the luciferase activity of each group was examined by GloMax Navigator (Promega), and three biological repeats from each group were used for statistical analysis.

### Antibodies and small molecules

The primary antibodies used in this study include anti-HEXIM1 (WB:1:5,000; IF:1:200; IP:1:100, Bethyl, A303-112A-T), anti-GFP (WB:1:3,000, Proteintech, 66002-1-Ig), anti-HA (WB:1:10,000; IP:1:100, Proteintech, 66006-2-Ig), anti-Flag (WB:1:5,000; IP:1:200; IF:1:1,000, Proteintech, 66008-4-Ig); anti-GAPDH (WB:1:5,000, Proteintech, 66031-1-Ig), anti-MYC (WB:1:5,000, Proteintech, 60003-2-Ig); anti-β-tubulin (WB:1:1,000, Abmart, M30109), anti-RNA polymerase II CTD repeat YSPTSPS (phospho S2) (WB:1:1,000; IF:1:200; CUT&Tag:1:50, Abcam, ab5095), anti-phospho-CDK9 T186 (WB:1:1,500, Abclonal, AP0810), anti-CDK9 (WB:1:1,500; IF:1:200; IP:1:100, Abclonal, A11145), anti-ICP4 (WB:1:700; IF:1:200, Virusys), anti-ICP22 (WB:1:500Virusys), anti-pUL47 (WB:1:500, Virusys), and anti-ICP8 (WB:1:500, Virusys). HRP-conjugated AffiniPure goat anti-mouse IgG (H + L) (SA00001-1) and HRP-conjugated AffiniPure goat anti-rabbit IgG (H + L) (SA00001-2) were purchased from Proteintech (WB: 1:5,000). Alexa Fluor 488-conjugated goat anti-rabbit IgG (H + L) secondary antibody (A11008) and Alexa Fluor 568-conjugated goat anti-mouse IgG (H + L) secondary antibody (A11004) were purchased from Thermo Fisher Scientific (IF: 1:1,000). The drug HMBA was purchased from TargetMol (T7863).

### Animal experiment

Ducklings were reared for 14 days in a suitable feeding environment and inoculated with 10^5^ TCID50 AnHV-1 via intramuscular injection. An equal volume of phosphate-buffered saline (PBS) was intramuscularly injected into ducks as a control. Six ducklings were randomly selected from each group on the first, third, and fifth days after infection. Organs were collected and labeled in a clean sampling bag and stored at −80°C (Thermo Fisher Scientific).

### Sequence alignment

Nucleotide and amino acid sequences were downloaded from NCBI (https://www.ncbi.nlm.nih.gov/) and aligned using the BLAST program (https://blast.ncbi.nlm.nih.gov/Blast.cgi) and DNAMAN (version 6.0.3.99).

*Anas platyrhynchos* (mallard)-HEXIM1 gene ID: 101802465; *Gallus gallus* (chicken)-HEXIM1 gene ID: 100857796; *Homo sapiens* (human)-HEXIM1 gene ID: 10614; *Mus musculus* (house mouse)-HEXIM1 gene ID: 192231; *Sus scrofa* (pig)-HEXIM1 gene ID: 1005207630; *Human alphaherpesvirus 1*-US1 gene ID: 2703435; *Anatid alphaherpesvirus 1*-US1 gene ID: 822340.

### RNA-seq

DEFs were seeded in a 24-well dish with mock-infected or AnHV-1 cells at an MOI of 1. At 12 hpi, total RNA was extracted using the RNA-easy Isolation Reagent (Vazyme, R701-01) according to the manufacturer’s recommendations. RNA purity and quantification were assessed using the NanoDrop 2000 spectrophotometer (Thermo Scientific, USA). RNA integrity was evaluated using an Agilent 2100 Bioanalyzer (Agilent Technologies, Santa Clara, CA, USA). Libraries were constructed using a TruSeq Stranded mRNA LT Sample Prep Kit (Illumina, San Diego, CA, USA) according to the manufacturer’s instructions. The transcriptome sequencing and analysis were conducted by OE Biotech Co., Ltd. (Shanghai, China).

The libraries were sequenced on an Illumina sequencing platform (HiSeqTM 2500 or Illumina HiSeq X Ten), and 125 bp/150 bp paired-end reads were generated. About 50 million raw reads for each sample were generated. Raw reads of fastq format were first processed using fastp, and the low-quality reads were removed to obtain the clean reads for subsequent analyses. The clean reads were mapped to the duck genome (GCF_015476345.1) and Anatid herpesvirus 1 (GCF_000885795.1) using HISAT2. FPKM of each gene was calculated, and the read counts of each gene were obtained by HTSeq-count. PCA analysis was performed using R (v 3.2.0) to evaluate the biological duplication of samples.

Differential expression analysis was performed using the DESeq2. *Q* value < 0.05 and foldchange > 1.5 or foldchange < 0.67 were set as the threshold for significantly differentially expressed genes (DEGs). Hierarchical cluster analysis of DEGs was performed using R (v 3.2.0) to demonstrate the expression pattern of genes in different groups and samples. The radar map of the top 30 genes was drawn to show the expression of upregulated or downregulated DEGs using R package gradeR. Gene Set Enrichment Analysis was performed using GSEA software. The analysis used a predefined gene set, and the genes were ranked according to the degree of differential expression in the two types of samples. Then, it was tested whether the predefined gene set was enriched at the top or bottom of the ranking list.

### RT-qPCR

#### DEF samples

Total RNA was isolated from the cells using RNA-easy Isolation Reagent (Vazyme, R701-01) according to the manufacturer’s instructions. The RNA was reverse transcribed with SuperMix for qPCR (gDNA digester plus) (Yeasen, 11141ES60), and cDNA was quantified with gene-specific primer pairs (Table 1). qPCRs were conducted in a CFX96 system (Bio-Rad) using qPCR SYBR Green Master Mix (Yeasen, 11184ES08), and reactions were denatured at 95°C for 30 s, followed by 35 two-step cycles of 95°C for 5 s and 60°C for 30 s. The relative gene expression levels were normalized against that of 18S rRNA.

#### Tissue samples

Tissue samples were placed in RNA-easy Isolation Reagent (Vazyme, R701-01) and homogenized (M.P. Biomedicals, FastPrep-24), and RNA was extracted according to the reagent instructions. The reverse transcription steps were the same as those for the cell samples.

Primers were designed using Primer Premier (version 5.0), visual annotation analysis of nucleotide and amino acid sequences was performed using SnapGene (version 2.3.2), and analysis of real-time qPCR results was performed using Bio-Rad CFX Manager (version 3.1).

### Western blotting

Samples were washed twice with ice-cold PBS and lysed with RIPA containing a protease inhibitor cocktail (Beyotime, P0013B). The resulting proteins were separated via 8% SDS-PAGE and then transferred to a PVDF membrane (Bio-Rad, 1620184). The membrane was blocked with 5% skim milk powder (Sangon Biotech, A600669) at RT for 3 h and incubated overnight (4°C) with primary antibodies. Goat anti-rabbit IgG (1:5,000) or goat anti-mouse IgG (1:5,000) was used as the secondary antibody and incubated with the blot for 1 h at 37°C. The proteins were visualized using Clarity Western ECL substrate (Bio-Rad). The graphs were stored in Image Lab, Bio-Rad. The expression of β-tubulin and GAPDH, which were used as loading controls, was detected with mouse anti-β-tubulin antibody and mouse anti-GAPDH antibody. The gray values of each band in western blot were analyzed by Image J and compared with the gray values of the corresponding reference proteins after normalization to the control group.

### Coimmunoprecipitation

After transfection or infection at a specified time point, the cells were lysed with IP lysis buffer (Beyotime, P0013). A portion of the total lysate was retained as a whole-cell extract. The rest of the lysate supernatant was divided into two samples and incubated with rabbit control IgG (Abclonal, AC005) or mouse control IgG (Abclonal, AC011) and target antibodies at 4°C for 24 h. Then, every sample was incubated with protein A + G Magnetic Beads (Beyotime, P2108) at RT for 1 h. The protein A + G Magnetic Beads were intensively washed with 1× TBS three times. Finally, all samples were discarded with the supernatants, and 50 µL of PBS and 12 µL of 5× loading buffer were added. The subsequent steps were the same as those used for western blotting.

### Immunofluorescence

Cells grown on glass coverslips in a 24-well plate were fixed in 4% paraformaldehyde (Keshi, 30525-89-4) for 20 min, permeabilized with 0.2% Triton X-100 (in PBS, Solarbio, T8200) for 20 min, and then blocked with 5% BSA (in PBS, Beyotime, ST023) for 2 h at room temperature (RT). Primary antibodies were incubated with cells overnight at 4°C and then with Alexa Fluor-conjugated secondary antibodies for 1 h at 37°C. Next, the cells were counterstained with DAPI (4′,6′-diamidino-2-phenylindole, 1:100; Solarbio, C0060) in the dark for 10 min to visualize the nuclei. Finally, the tablets were sealed using an anti-fluorescence quenching agent (Beyotime, P0128S).

Images were captured with a confocal laser scanning microscope at 400× magnification (eyepiece 10×, objective 40×). Photo software: OlyVIA; the fluorescence microscope (80i) and microscope lens (including color filter, E400) were from Nikon, Japan. The software used for localization statistics was ImageJ.

### Cell viability

Cell Counting Kit (CCK-8) (Yeasen, 40203ES60) was used to detect whether different concentrations of HMBA affected DEF viability according to the manufacturer’s instructions.

### Virus growth analysis

To evaluate the effect of HEXIM1 on viral growth, DEFs seeded in 48-well plate were treated with HMBA/siRNA/p-HEXIM1 before infection. To quickly assess the overall effect of HEXIM1 on AnHV-1 replication, a reporter virus of AnHV-1 with an EGFP cassette, which showed no obvious difference compared to the WT virus, has been inoculated into DEF cells under the condition of HEXIM1 over-expression or knockdown. The fluorescence was observed and recorded under the microscope (Nikon, Japan) at the indicated time points. The cells infected with AnHV-1 at an MOI of 0.01 were collected at various points after infection to determine progeny virus using the Muench and Reed method. The viral titer was determined based on three biological repeats.

### CUT&Tag

DEFs seeded in a 6-well plate were transfected with siRNA and infected with AnHV-1 at an MOI of 0.1 after 4 h. The detailed operation was according to Hieff NGS G-Type *In Situ* DNA Binding Profiling Library Prep Kit for Illumina CUT&Tag Kit (Yeasen, 12598ES12). Simply, after 18 h, cells were counted, harvested, and centrifuged for 3 min at 600 × *g* at room temperature. Cells were washed twice in wash buffer (protease inhibitor cocktail, EDTA-free) by gentle pipetting. ConA-coated magnetic beads were washed twice in ConA binding buffer by gentle pipetting, and 10 µL of activated beads was added and incubated with each sample at RT for 10 min. We observed that binding cells to the beads in this step increased binding efficiency. The unbound supernatant was removed, and bead-bound cells were resuspended in 49 µL of cell wash buffer (+) (protease inhibitor cocktail, EDTA-free; 5% digitonin) and a 1:50 dilution of the appropriate primary antibody (anti-RNA polymerase II CTD repeat YSPTSPS phospho S2, Abcam, ab5095). Primary antibody incubation was performed on a rotating platform for 2 h at room temperature. The primary antibody was removed by placing the tube on a magnet stand, and an appropriate secondary antibody (such as goat anti-rabbit IgG for a rabbit primary antibody, Abclonal, AS070) was diluted 1:100 in 49.5 µL of cell wash buffer (+) (protease inhibitor cocktail, EDTA-free; 5% digitonin), and cells were incubated at RT for 45 min. Cells were washed twice using the magnet stand in 65 µL of cell wash buffer (+) to remove unbound antibodies. A 1:50 dilution of pA/G-Transposome Mix was prepared in Tag Buffer (5% digitonin; 3 M NaCl; protease inhibitor cocktail, EDTA-free). After the removal of the liquid, while the sample was on the magnet stand, 50 µL was added to the cells with gentle vortexing, followed by incubation with pA/G-Transposome Mix at RT for 1 h. Cells were washed 3× for 2 min in 100 µL of Tag Buffer to remove unbound pA/G protein. Next, the cells were resuspended in 30 µL of Tag buffer with 1 µL 30× activating buffer and incubated at 37°C (Bioscience Company) for 1 h. To stop tagmentation, 2 µL of 15× termination solution, 1 µL of DNA spike-in mix, and 1 µL of 30× proteinase K were added to 30 µL of sample, which was incubated at 55°C in a thermal cycler (Bio-Rad, T100) for 30 min. Tubes were placed on a magnet stand to clear, and then, the liquid was carefully withdrawn (30 µL). To extract the DNA, 40 µL of DNA selection beads was added to each tube with vortexing, quickly spun, and held for 5 min. Tubes were placed on a magnet stand to clear, and then, the liquid was carefully withdrawn. Without disturbing the beads, the beads were washed twice in 200 µL of 80% ethanol. After allowing the samples to dry for ~3 min, 21 µL of ddH_2_O was added, and the tubes were vortexed, quickly spun, and allowed to sit for 5 min. Tubes were placed on a magnet stand, and the liquid was withdrawn and transferred to a fresh tube.

To amplify libraries, 20 µL of DNA was mixed with 1 µL of a universal N5 primer and a uniquely barcoded N7 primer using a different barcode for each sample. A volume of 25 µL of 2× Ultima Amplification mix and PCR Primer Mix were added and mixed. The sample was placed in a thermal cycler (Bio-Rad, T100) with a heated lid and subjected to the following cycling conditions: 72°C for 3 min; 95°C for 30 s; 10 cycles of 95°C for 10 s, 55°C for 30 s, and 72°C for 30 s; final extension at 72°C for 5 min and hold at 4°C. Post-PCR clean-up was performed by adding 1.2× DNA selection beads, and the following steps were the same as the first time to add DNA selection beads.

The final samples were sent to the company for high-throughput sequencing using the IlluminaS platform. The returned samples were used for RT-qPCR (Bio-Rad, CFX Crmect) to further verify the sequencing results. The primers used for verification were designed in the coding sequence (CDS) region of corresponding genes (Table 1).

### Data processing of CUT&Tag

The standard analysis was supplied by the CUT&Tag sequencing company (Romics, Shanghai, China). Principally, Trimmomatic software was used to remove the adapter and low-quality reads. Quality distribution plot and base content distribution were generated by FastQC. Before read mapping, clean reads were obtained from the raw reads by removing the adapter sequences. The clean reads were then aligned to reference genome sequences using the bwa program. We calculate the fragment sizes for read pairs given a BAM file from paired-end sequencing. Several regions were sampled depending on the size of the genome and the number of processors to estimate the summary statistics on the fragment lengths. Properly paired reads were used for computation. The bam file was generated by the unique mapped reads as an input file, using macs2 software for callpeak with cutoff *q* value <0.05. Reads distributions (from bigwig) across genes and peaks are presented as an average plot of box plot and curve plot (average of read signals across all genes). The deeptools tool is used for this analysis. The results of the annotations were counted, and the distribution results were plotted using the function plotAnnoPie of ChIPseeker.

### Statistical analysis

The RT-qPCR, western blotting, immunofluorescence, virus growth analysis, dual luciferase reporter assay, and viral genome determination results were analyzed using GraphPad Prism software (version 7.0) for biological statistics (*t* test), mapping, and analysis. The statistically significant differences are indicated as follows: *P < 0.05; **P < 0.01; ***P < 0.001; ****P < 0.0001; and ns, not significant.

## Data Availability

Raw RNA-Seq and CUT&Tag data are available in the Sequence Read Archive (SRA) public database under accession no. PRJNA1072040 and PRJNA1071732. .

## SUPPLEMENTAL MATERIAL

Supplemental table legends jvi.01392-23-s0001.docx Legends for Tables S1 to S3.

Supplemental figure legends jvi.01392-23-s0002.docx Legends for Fig. S1 to S5.

Fig. S1 jvi.01392-23-s0003.tif Multiple siRNA knockdown of HEXIM1 decreases AnHV-1 viral gene expression.

Fig. S2 jvi.01392-23-s0004.tif The knockdown of HEXIM1 had no influence on the promoter activity of IFNβ.

Fig. S3 jvi.01392-23-s0005.tif AnHV-1 replication and viral gene expression are aided by HMBA-induced HEXIM1 expression.

Fig. S4 jvi.01392-23-s0006.tif The effect of HEXIM1 on CDK9 and RNAPII S2 phosphorylation and CDK9-HEXIM1 interaction.

Fig. S5 jvi.01392-23-s0007.tif Influence of HEXIM1 on the proliferation of WT and US1-mutant.

Table S1 jvi.01392-23-s0008.xlsx Results of GSEA analysis of RNA-Seq.

Table S2 jvi.01392-23-s0009.xlsx Analysis of differentially expressed genes obtained by RNA-Seq.

Table S3 jvi.01392-23-s0010.xlsx Difference peak annotation analysis of CUT&Tag result.

ASM does not own the copyrights to Supplemental Material that may be linked to, or accessed through, an article. The authors have granted ASM a non-exclusive, world-wide license to publish the Supplemental Material files. Please contact the corresponding author directly for reuse.

## References

[1] Pellett P, Davison AJ, Eberle R, Ehlers B, Hayward GS, Lacoste V, Minson AC, Nicholas J, Roizman B, Studdert M, Wang F. 2011. Herpesvirales, p 99–107. In Virus taxonomy, 9th report of the international committee on taxonomy of viruses

[2] Scherer J, Enquist LW. 2017. Alphaherpesviruses: parasites of the peripheral nervous system. Future Virol 12:555–559. doi:10.2217/fvl-2017-0082

[3] Aayesha Riaz M-u-H, Kifayatullah NA. 2017. Recent understanding of the classification and life cycle of herpesviruses: a review. Science letters.

[4] Dremel SE, DeLuca NA. 2019. Genome replication affects transcription factor binding mediating the cascade of herpes simplex virus transcription. Proc Natl Acad Sci U S A 116:3734–3739. doi:10.1073/pnas.1818463116 30808759 PMC6397523

[5] Gruffat H, Marchione R, Manet E. 2016. Herpesvirus late gene expression: a viral-specific pre-initiation complex is key. Front Microbiol 7:869. doi:10.3389/fmicb.2016.00869 27375590 PMC4893493

[6] Bauer DLV, Tellier M, Martínez-Alonso M, Nojima T, Proudfoot NJ, Murphy S, Fodor E. 2018. Influenza virus mounts a two-pronged attack on host RNA polymerase II transcription. Cell Rep 23:2119–2129. doi:10.1016/j.celrep.2018.04.047 29768209 PMC5972227

[7] Xiao K, Xiong D, Chen G, Yu J, Li Y, Chen K, Zhang L, Xu Y, Xu Q, Huang X, Gao A, Cao K, Yan K, Dai J, Hu X, Ruan Y, Fu Z, Li G, Cao G. 2021. RUNX1-mediated alphaherpesvirus-host trans-species chromatin interaction promotes viral transcription. Sci Adv 7:eabf8962. doi:10.1126/sciadv.abf8962 34162542 PMC8221632

[8] Hennig T, Djakovic L, Dölken L, Whisnant AW. 2021. A review of the multipronged attack of herpes simplex virus 1 on the host transcriptional machinery. Viruses 13:1836. doi:10.3390/v13091836 34578417 PMC8473234

[9] Chen CP, Lyu Y, Chuang F, Nakano K, Izumiya C, Jin D, Campbell M, Izumiya Y. 2017. Kaposi's sarcoma-associated herpesvirus hijacks RNA polymerase II to create a viral transcriptional factory. J Virol 91:e02491-16. doi:10.1128/JVI.02491-16 28331082 PMC5432858

[10] Cramer P. 2019. Eukaryotic transcription turns 50. Cell 179:808–812. doi:10.1016/j.cell.2019.09.018 31675494

[11] Phatnani HP, Greenleaf AL. 2006. Phosphorylation and functions of the RNA polymerase II CTD. Genes Dev 20:2922–2936. doi:10.1101/gad.1477006 17079683

[12] Liu X, Bushnell DA, Kornberg RD. 2013. RNA polymerase II transcription: structure and mechanism. Biochim Biophys Acta 1829:2–8. doi:10.1016/j.bbagrm.2012.09.003 23000482 PMC4244541

[13] Harlen KM, Churchman LS. 2017. The code and beyond: transcription regulation by the RNA polymerase II carboxy-terminal domain. Nat Rev Mol Cell Biol 18:263–273. doi:10.1038/nrm.2017.10 28248323

[14] Chapman RD, Heidemann M, Hintermair C, Eick D. 2008. Molecular evolution of the RNA polymerase II CTD. Trends Genet 24:289–296. doi:10.1016/j.tig.2008.03.010 18472177

[15] Schier AC, Taatjes DJ. 2020. Structure and mechanism of the RNA polymerase II transcription machinery. Genes Dev 34:465–488. doi:10.1101/gad.335679.119 32238450 PMC7111264

[16] Suh H, Ficarro SB, Kang UB, Chun Y, Marto JA, Buratowski S. 2016. Direct analysis of phosphorylation sites on the Rpb1 C-terminal domain of RNA polymerase II. Mol Cell 61:297–304. doi:10.1016/j.molcel.2015.12.021 26799764 PMC4724063

[17] Hsin JP, Xiang K, Manley JL. 2014. Function and control of RNA polymerase II C-terminal domain phosphorylation in vertebrate transcription and RNA processing. Mol Cell Biol 34:2488–2498. doi:10.1128/MCB.00181-14 24752900 PMC4054314

[18] Eick D, Geyer M. 2013. The RNA polymerase II carboxy-terminal domain (CTD) code. Chem Rev 113:8456–8490. doi:10.1021/cr400071f 23952966

[19] Brogie JE, Price DH. 2017. Reconstitution of a functional 7SK snRNP. Nucleic Acids Res 45:6864–6880. doi:10.1093/nar/gkx262 28431135 PMC5499737

[20] Diribarne G, Bensaude O. 2009. 7SK RNA, a non-coding RNA regulating P-TEFb, a general transcription factor. RNA Biol 6:122–128. doi:10.4161/rna.6.2.8115 19246988

[21] Peterlin BM, Price DH. 2006. Controlling the elongation phase of transcription with P-TEFb. Mol Cell 23:297–305. doi:10.1016/j.molcel.2006.06.014 16885020

[22] Michels AA, Bensaude O. 2018. Hexim1, an RNA-controlled protein hub. Transcription 9:262–271. doi:10.1080/21541264.2018.1429836 29345523 PMC6104690

[23] Chen FX, Smith ER, Shilatifard A. 2018. Born to run: control of transcription elongation by RNA polymerase II. Nat Rev Mol Cell Biol 19:464–478. doi:10.1038/s41580-018-0010-5 29740129

[24] Quaresma CAJ, Bugai A, Barboric M. 2016. Cracking the control of RNA polymerase II elongation by 7SK snRNP and P-TEFb. Nucleic Acids Res 44:7527–7539. doi:10.1093/nar/gkw585 27369380 PMC5027500

[25] Kobbi L, Demey-Thomas E, Braye F, Proux F, Kolesnikova O, Vinh J, Poterszman A, Bensaude O. 2016. An evolutionary conserved Hexim1 peptide binds to the Cdk9 catalytic site to inhibit P-TEFb. Proc Natl Acad Sci U S A 113:12721–12726. doi:10.1073/pnas.1612331113 27791144 PMC5111705

[26] Fujinaga K, Barboric M, Li Q, Luo Z, Price DH, Peterlin BM. 2012. PKC phosphorylates HEXIM1 and regulates P-TEFb activity. Nucleic Acids Res 40:9160–9170. doi:10.1093/nar/gks682 22821562 PMC3467075

[27] Devaraj SGT, Fiskus W, Shah B, Qi J, Sun B, Iyer SP, Sharma S, Bradner JE, Bhalla KN. 2016. HEXIM1 induction is mechanistically involved in mediating anti-AML activity of BET protein bromodomain antagonist. Leukemia 30:504–508. doi:10.1038/leu.2015.142 26148704 PMC4809433

[28] Chen R, Yik JHN, Lew QJ, Chao S-H. 2014. Brd4 and HEXIM1: multiple roles in P-TEFb regulation and cancer. Biomed Res Int 2014:232870. doi:10.1155/2014/232870 24592384 PMC3925632

[29] Zhang J, Li G, Ye X. 2010. Cyclin T1/CDK9 interacts with influenza A virus polymerase and facilitates its association with cellular RNA polymerase II. J Virol 84:12619–12627. doi:10.1128/JVI.01696-10 20943989 PMC3004352

[30] Vijayalingam S, Chinnadurai G. 2013. Adenovirus L-E1A activates transcription through mediator complex-dependent recruitment of the super elongation complex. J Virol 87:3425–3434. doi:10.1128/JVI.03046-12 23302885 PMC3592126

[31] Zaborowska J, Isa NF, Murphy S. 2016. P-TEFb goes viral. Bioessays 38:S75–S85. doi:10.1002/bies.201670912 27417125

[32] Lukarska M, Fournier G, Pflug A, Resa-Infante P, Reich S, Naffakh N, Cusack S. 2017. Structural basis of an essential interaction between influenza polymerase and Pol II CTD. Nature 541:117–121. doi:10.1038/nature20594 28002402

[33] Zaborowska J, Baumli S, Laitem C, O’Reilly D, Thomas PH, O’Hare P, Murphy S. 2014. Herpes simplex virus 1 (HSV-1) ICP22 protein directly interacts with cyclin-dependent kinase (CDK)9 to inhibit RNA polymerase II transcription elongation. PLoS One 9:e107654. doi:10.1371/journal.pone.0107654 25233083 PMC4169428

[34] Guo L, Wu W, Liu L, Wang L, Zhang Y, Wu L, Guan Y, Li Q. 2012. Herpes simplex virus 1 ICP22 inhibits the transcription of viral gene promoters by binding to and blocking the recruitment of P-TEFb. PLoS One 7:e45749. doi:10.1371/journal.pone.0045749 23029222 PMC3454370

[35] Chang PC, Li M. 2008. Kaposi's sarcoma-associated herpesvirus K-cyclin interacts with Cdk9 and stimulates Cdk9-mediated phosphorylation of p53 tumor suppressor. J Virol 82:278–290. doi:10.1128/JVI.01552-07 17942552 PMC2224387

[36] Palermo RD, Webb HM, West MJ. 2011. RNA polymerase II stalling promotes nucleosome occlusion and pTEFb recruitment to drive immortalization by Epstein-Barr virus. PLoS Pathog 7:e1002334. doi:10.1371/journal.ppat.1002334 22046134 PMC3203192

[37] Wimmer J, Fujinaga K, Taube R, Cujec TP, Zhu Y, Peng J, Price DH, Peterlin BM. 1999. Interactions between Tat and TAR and human immunodeficiency virus replication are facilitated by human cyclin T1 but not cyclins T2a or T2b. Virology 255:182–189. doi:10.1006/viro.1998.9589 10049833

[38] Wei P, Garber ME, Fang SM, Fischer WH, Jones KA. 1998. A novel CDK9-associated C-type cyclin interacts directly with HIV-1 Tat and mediates its high-affinity, loop-specific binding to TAR RNA. Cell 92:451–462. doi:10.1016/s0092-8674(00)80939-3 9491887

[39] Barboric M, Yik JHN, Czudnochowski N, Yang Z, Chen R, Contreras X, Geyer M, Matija Peterlin B, Zhou Q. 2007. Tat competes with HEXIM1 to increase the active pool of P-TEFb for HIV-1 transcription. Nucleic Acids Res 35:2003–2012. doi:10.1093/nar/gkm063 17341462 PMC1874611

[40] Faust TB, Li Y, Bacon CW, Jang GM, Weiss A, Jayaraman B, Newton BW, Krogan NJ, D’Orso I, Frankel AD. 2018. The HIV-1 Tat protein recruits a ubiquitin ligase to reorganize the 7SK snRNP for transcriptional activation. Elife 7:e31879. doi:10.7554/eLife.31879 29845934 PMC5999396

[41] Wu Y, Cheng A, Wang M, Zhu D, Jia R, Chen S, Zhou Y, Chen X. 2012. Comparative genomic analysis of duck enteritis virus strains. J Virol 86:13841–13842. doi:10.1128/JVI.01517-12 23166249 PMC3503134

[42] Stoute ST, Metwally SA, Cheng A, Guérin JL, Palya VJ. 2020. Diseases of poultry

[43] Yik JHN, Chen R, Nishimura R, Jennings JL, Link AJ, Zhou Q. 2003. Inhibition of P-TEFb (CDK9/Cyclin T) kinase and RNA polymerase II transcription by the coordinated actions of HEXIM1 and 7SK snRNA. Mol Cell 12:971–982. doi:10.1016/s1097-2765(03)00388-5 14580347

[44] Barboric M, Kohoutek J, Price JP, Blazek D, Price DH, Peterlin BM. 2005. Interplay between 7SK snRNA and oppositely charged regions in HEXIM1 direct the inhibition of P-TEFb. EMBO J 24:4291–4303. doi:10.1038/sj.emboj.7600883 16362050 PMC1356324

[45] Michels AA, Fraldi A, Li Q, Adamson TE, Bonnet F, Nguyen VT, Sedore SC, Price JP, Price DH, Lania L, Bensaude O. 2004. Binding of the 7SK snRNA turns the HEXIM1 protein into a P-TEFb (CDK9/cyclin T) inhibitor. EMBO J 23:2608–2619. doi:10.1038/sj.emboj.7600275 15201869 PMC449783

[46] Dey A, Chao SH, Lane DP. 2007. HEXIM1 and the control of transcription elongation: from cancer and inflammation to AIDS and cardiac hypertrophy. Cell Cycle 6:1856–1863. doi:10.4161/cc.6.15.4556 17671421

[47] Blazek D, Barboric M, Kohoutek J, Oven I, Peterlin BM. 2005. Oligomerization of HEXIM1 via 7SK snRNA and coiled-coil region directs the inhibition of P-TEFb. Nucleic Acids Res 33:7000–7010. doi:10.1093/nar/gki997 16377779 PMC1322273

[48] Bigalke JM, Dames SA, Blankenfeldt W, Grzesiek S, Geyer M. 2011. Structure and dynamics of a stabilized coiled-coil domain in the P-TEFb regulator Hexim1. J Mol Biol 414:639–653. doi:10.1016/j.jmb.2011.10.022 22033481

[49] Alfonso-Dunn R, Arbuckle JH, Vogel JL, Kristie TM. 2020. Inhibition of the super elongation complex suppresses herpes simplex virus immediate early gene expression, lytic infection, and reactivation from latency. mBio 11:e01216-20. doi:10.1128/mBio.01216-20 32518191 PMC7373197

[50] Gao L, Liu R, Yang F, Li X, Liu C, Qi X, Cui H, Zhang Y, Wang S, Wang X, Gao Y, Li K. 2022. Duck enteritis virus inhibits the cGAS-STING DNA-sensing pathway to evade the innate immune response. J Virol 96:e0157822. doi:10.1128/jvi.01578-22 36448809 PMC9769366

[51] Darwish AS, Grady LM, Bai P, Weller SK. 2015. ICP8 filament formation is essential for replication compartment formation during herpes simplex virus infection. J Virol 90:2561–2570. doi:10.1128/JVI.02854-15 26676794 PMC4810729

[52] Yiu SPT, Guo R, Zerbe C, Weekes MP, Gewurz BE. 2022. Epstein-Barr virus BNRF1 destabilizes SMC5/6 cohesin complexes to evade its restriction of replication compartments. Cell Rep 38:110411. doi:10.1016/j.celrep.2022.110411 35263599 PMC8981113

[53] Taylor TJ, McNamee EE, Day C, Knipe DM. 2003. Herpes simplex virus replication compartments can form by coalescence of smaller compartments. Virology 309:232–247. doi:10.1016/s0042-6822(03)00107-7 12758171

[54] Chang L, Godinez WJ, Kim I-H, Tektonidis M, de Lanerolle P, Eils R, Rohr K, Knipe DM. 2011. Herpesviral replication compartments move and coalesce at nuclear speckles to enhance export of viral late mRNA. Proc Natl Acad Sci U S A 108:E136–E144. doi:10.1073/pnas.1103411108 21555562 PMC3102408

[55] Knipe DM, Senechek D, Rice SA, Smith JL. 1987. Stages in the nuclear association of the herpes simplex virus transcriptional activator protein ICP4. J Virol 61:276–284. doi:10.1128/JVI.61.2.276-284.1987 3027360 PMC253947

[56] Rice SA, Long MC, Lam V, Spencer CA. 1994. RNA polymerase II is aberrantly phosphorylated and localized to viral replication compartments following herpes simplex virus infection. J Virol 68:988–1001. doi:10.1128/JVI.68.2.988-1001.1994 8289400 PMC236537

[57] Xu H, Wang J, Deng Y, Hou F, Fu Y, Chen S, Zou W, Pan D, Chen B. 2022. Two-color CRISPR imaging reveals dynamics of herpes simplex virus 1 replication compartments and virus-host interactions. J Virol 96:e0092022. doi:10.1128/jvi.00920-22 36453882 PMC9769385

[58] Isa NF, Bensaude O, Aziz NC, Murphy S. 2021. HSV-1 ICP22 is a selective viral repressor of cellular RNA polymerase II-mediated transcription elongation. Vaccines (Basel) 9:1054. doi:10.3390/vaccines9101054 34696162 PMC8539892

[59] Xue Y, Yang Z, Chen R, Zhou Q. 2010. A capping-independent function of MePCE in stabilizing 7SK snRNA and facilitating the assembly of 7SK snRNP. Nucleic Acids Res 38:360–369. doi:10.1093/nar/gkp977 19906723 PMC2811026

[60] Zhao Z, Tang KW, Muylaert I, Samuelsson T, Elias P. 2017. CDK9 and SPT5 proteins are specifically required for expression of herpes simplex virus 1 replication-dependent late genes. J Biol Chem 292:15489–15500. doi:10.1074/jbc.M117.806000 28743741 PMC5602406

[61] Fox HL, Dembowski JA, DeLuca NA. 2017. A herpesviral immediate early protein promotes transcription elongation of viral transcripts. mBio 8:e00745-17. doi:10.1128/mBio.00745-17 28611249 PMC5472187

[62] Ren K, Zhang W, Chen X, Ma Y, Dai Y, Fan Y, Hou Y, Tan RX, Li E. 2016. An epigenetic compound library screen identifies BET inhibitors that promote HSV-1 and -2 replication by bridging P-TEFb to viral gene promoters through BRD4. PLoS Pathog 12:e1005950. doi:10.1371/journal.ppat.1005950 27764245 PMC5072739

[63] Gurumurthy M, Tan CH, Ng R, Zeiger L, Lau J, Lee J, Dey A, Philp R, Li Q, Lim TM. 2008. Nucleophosmin interacts with HEXIM1 and regulates RNA polymerase II transcription. J Mol Biol 378:302–317. doi:10.1016/j.jmb.2008.02.055 18371977

[64] Perng Y-C, Campbell JA, Lenschow DJ, Yu D. 2014. Human cytomegalovirus pUL79 is an elongation factor of RNA polymerase II for viral gene transcription. PLoS Pathog 10:e1004350. doi:10.1371/journal.ppat.1004350 25166009 PMC4148446

[65] Bark-Jones SJ, Webb HM, West MJ. 2006. EBV EBNA 2 stimulates CDK9-dependent transcription and RNA polymerase II phosphorylation on serine 5. Oncogene 25:1775–1785. doi:10.1038/sj.onc.1209205 16314842

[66] Morchikh M, Cribier A, Raffel R, Amraoui S, Cau J, Severac D, Dubois E, Schwartz O, Bennasser Y, Benkirane M. 2017. HEXIM1 and NEAT1 long non-coding RNA form a multi-subunit complex that regulates DNA-mediated innate immune response. Mol Cell 67:387–399. doi:10.1016/j.molcel.2017.06.020 28712728

[67] Birkenheuer CH, Dunn L, Dufour R, Baines JD. 2022. ICP22 of herpes simplex virus 1 decreases RNA polymerase processivity. J Virol 96:e0219121. doi:10.1128/jvi.02191-21 35019725 PMC8906429

[68] Wu Y, Li Y, Wang M, Sun K, Jia R, Chen S, Zhu D, Liu M, Yang Q, Zhao X, Chen X, Cheng A. 2017. Preliminary study of the UL55 gene based on infectious Chinese virulent duck enteritis virus bacterial artificial chromosome clone. Virol J 14:78. doi:10.1186/s12985-017-0748-y 28407817 PMC5390382

