# Supplementary material for: An alpha-herpesvirus employs host HEXIM1 to promote viral transcription
Source: J Virol. 2024 Feb 16;98(3):e01392-23. doi: 10.1128/jvi.01392-23 (PMC10949456; doi:10.1128/jvi.01392-23)
Supplement: Supplemental table legends — Legends for Tables S1 to S3. [file jvi.01392-23-s0001.docx]

**Supplemental Table Legends**

**Tab. S1. Results of GSEA analysis of RNA-Seq.** Gene Set Enrichment Analysis (GSEA) of AnHV-1 and Mock infected DEF cells at 12 h.p.i were performed using GSEA software. The analysis was used a predefined gene set, and the genes sequenced by RNA-Seq were ranked according to the degree of differential expression in the two types of samples. The red marked gene sets were selected for plotting.

**Tab. S2.** **Analysis of differentially expressed genes obtained by RNA-Seq.** Differential expression analysis of AnHV-1 and Mock infected DEF cells at 12 h.p.i was performed using the DESeq2. Q value < 0.05 and foldchange > 1.5 or foldchange < 0.67 was set as the threshold for significantly differential expression gene (DEGs).

**Tab. S3. Difference Peak annotation analysis of CUT&Tag result.** We used the annotatePeak function of the R package ChIPseeker (Yu G, Wang L, He Q 2015) to annotate genomic features where peaks are located on the duck genome before and after AnHV-1 infection for 12 h. Functional regions annotated in this analysis include promoters, exons, UTR (untranslated regions, including 5’UTR and 3’UTR), introns, or Distal Intergenic regions.
