# Supplementary material for: An alpha-herpesvirus employs host HEXIM1 to promote viral transcription
Source: J Virol. 2024 Feb 16;98(3):e01392-23. doi: 10.1128/jvi.01392-23 (PMC10949456; doi:10.1128/jvi.01392-23)
Supplement: Supplemental figure legends — Legends for Fig. S1 to S5. [file jvi.01392-23-s0002.docx]

**Supplemental Figure Legends**

**Fig. S1. Multiple siRNA knockdown of HEXIM1 decrease AnHV-1 viral gene expression.** Influence of HEXIM1 knockdown on viral gene transcription (A), and protein expression (B). DEFs transfected with siNC or siHEXIM1-1/2 were infected with AnHV-1 at an MOI of 0.01. Cell lysates were collected at 24 hpi. The normalized gene expression in siNC cells was set to 1, and β-tubulin was used as the loading control. Data for A was collected using at least three biological replicates per sample. **, p<0.01; ***, p<0.001.

**Fig. S2.** **The knockdown of HEXIM1 had no influence on the promoter activity of IFNβ.** DEFs knockdown of HEXIM1 were transfected with pGL-IFNβ, pRL-TK, and the groups of pCAGGS-STING-MYC/pCAGGS/Mock/AnHV-1(1MOI) were for subsequent experiments. **A.** Flow chart for detecting the effect of siHEXIM1 on antiviral response(left), and the confirmation of HEXIM1 knockdown or STING expression by western blotting. GAPDH was used as the loading control. **B.** Dual luciferase reporter assay was used to detect the effect of siHEXIM1 on the activity of IFNβ promoter. STING was used as a positive control. Cell lysates were collected at 24 hpi. The data were performed with at least three biological replicates per sample; *, p < 0.05; ***, p<0.001; ns, not significant.

**Fig. S3.** **AnHV-1 replication and viral gene expression are aided by HMBA-induced HEXIM1 expression. A.** Chemical structure of HMBA. **B.** Effect of HMBA on cell viability and HEXIM1 expression. DEFs were treated for 24 hours with DMSO or HMBA at the indicated doses. The influence of HMBA concentrations on cell viability was investigated, and while RT-qPCR and Western blotting assay was used to identify the effect of HMBA concentrations on HEXIM1 mRNA and protein expression levels. In DMSO-treated cells, normalized gene expression was adjusted to 1, and GAPDH was employed as the loading control. **C.** Flow chart for detecting the effect of HEXIM1 overexpression on AnHV-1 replication and gene expression (up) and verification of HMBA-induced HEXIM1 induction through RT-qPCR (down). **D-G.** The overexpression effect of HEXIM1 on viral replication (D), titer (E), gene transcription (F) and protein expression (G). DEFs overexpressing HEXIM1 were infected with AnHV-1 at an MOI of 0.01. Cell lysates were collected at 24 hpi. The gray values of three repeated experiments were analyzed by Image J. The data on B, C, E, F, G were performed with at least three biological replicates per sample; *, p< 0.05; **, p<0.01; ***, p<0.001; ns, not significant.

**Fig. S4. The effect of HEXIM1 on CDK9 and RNAPII S2 phosphorylation, and CDK9-HEXIM1 interaction.** A. Western blotting analysis of corresponding host genes related to HEXIM1 at 24h.p.i. The normalized gene expression in pCAGGS- or siNC-treated cells was set to 1 and β-tubulin was used as the loading control. **B.** The effect of HEXIM1 overexpression on formation of CDK9-HEXIM1 complex.

**Fig. S5. Influence of HEXIM1 on the proliferation of WT and US1-mutant.** DEFs transfected with pCAGGS or pCAGGS-HEXIM1 were infected with AnHV-1 or AnHV-1 delUS1 at an MOI of 0.01. The data was performed on at least three biological replicates per sample. *, p < 0.05; **, p<0.01; ns, not significant.
