# Supplementary figures and images for: An alpha-herpesvirus employs host HEXIM1 to promote viral transcription
Source: J Virol. 2024 Feb 16;98(3):e01392-23. doi: 10.1128/jvi.01392-23 (PMC10949456; doi:10.1128/jvi.01392-23)

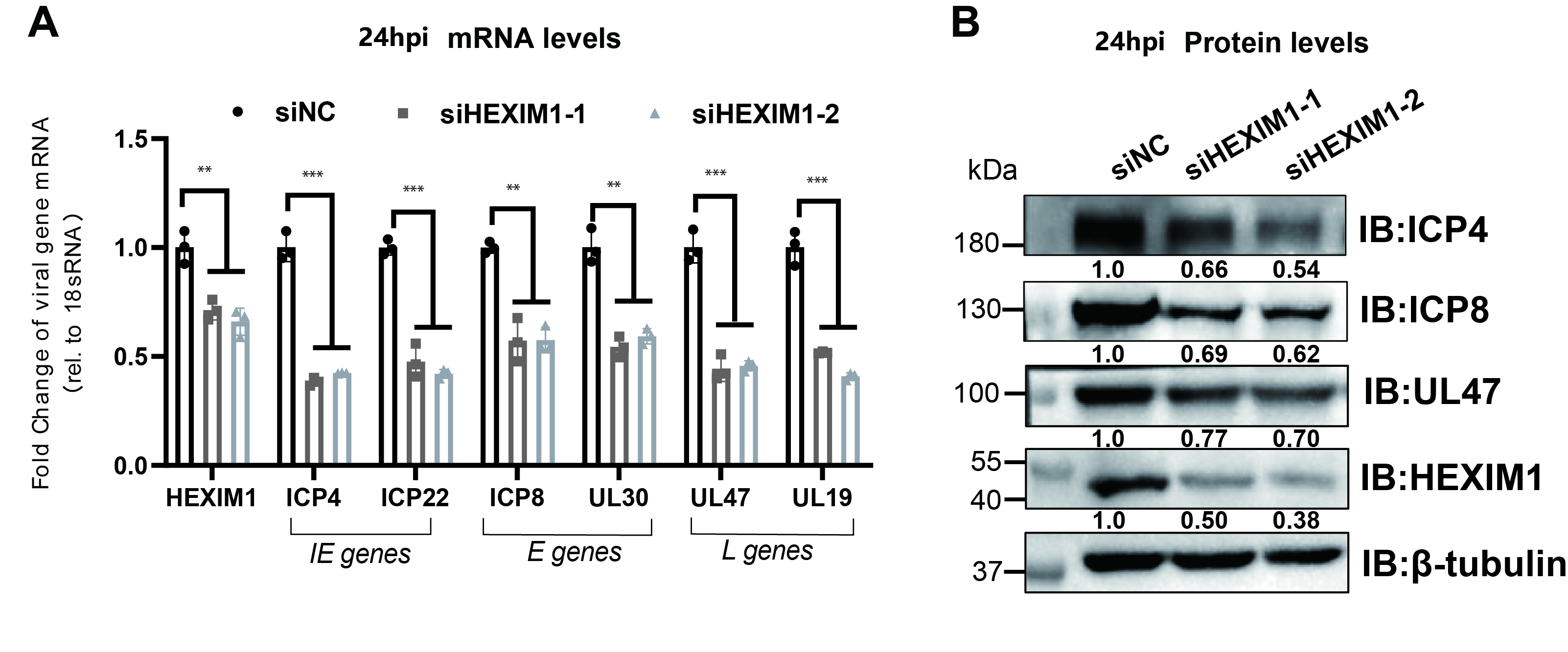

Supplement: Fig. S1 — Multiple siRNA knockdown of HEXIM1 decreases AnHV-1 viral gene expression. [file jvi.01392-23-s0003.tif]

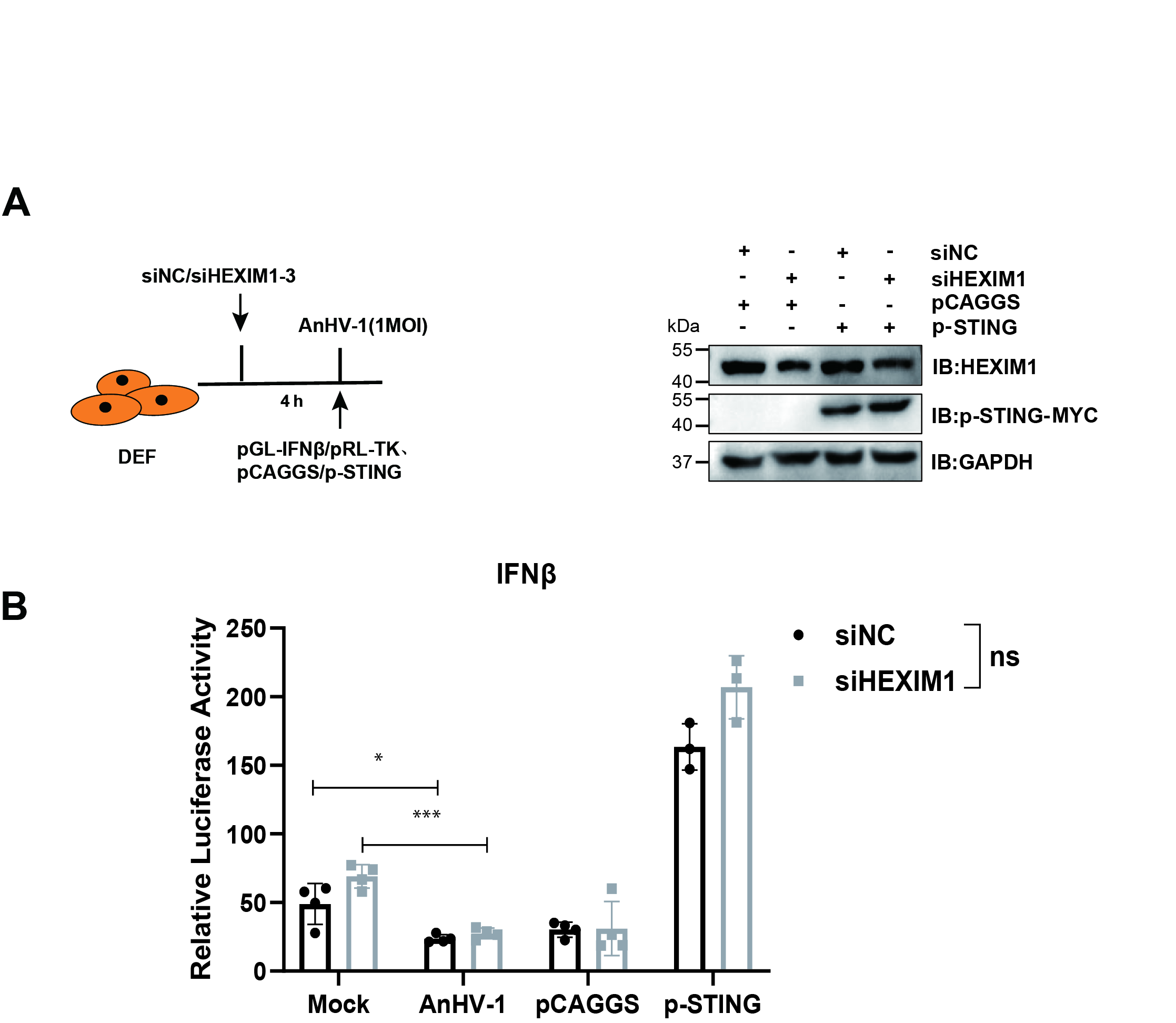

Supplement: Fig. S2 — The knockdown of HEXIM1 had no influence on the promoter activity of IFNβ. [file jvi.01392-23-s0004.tif]

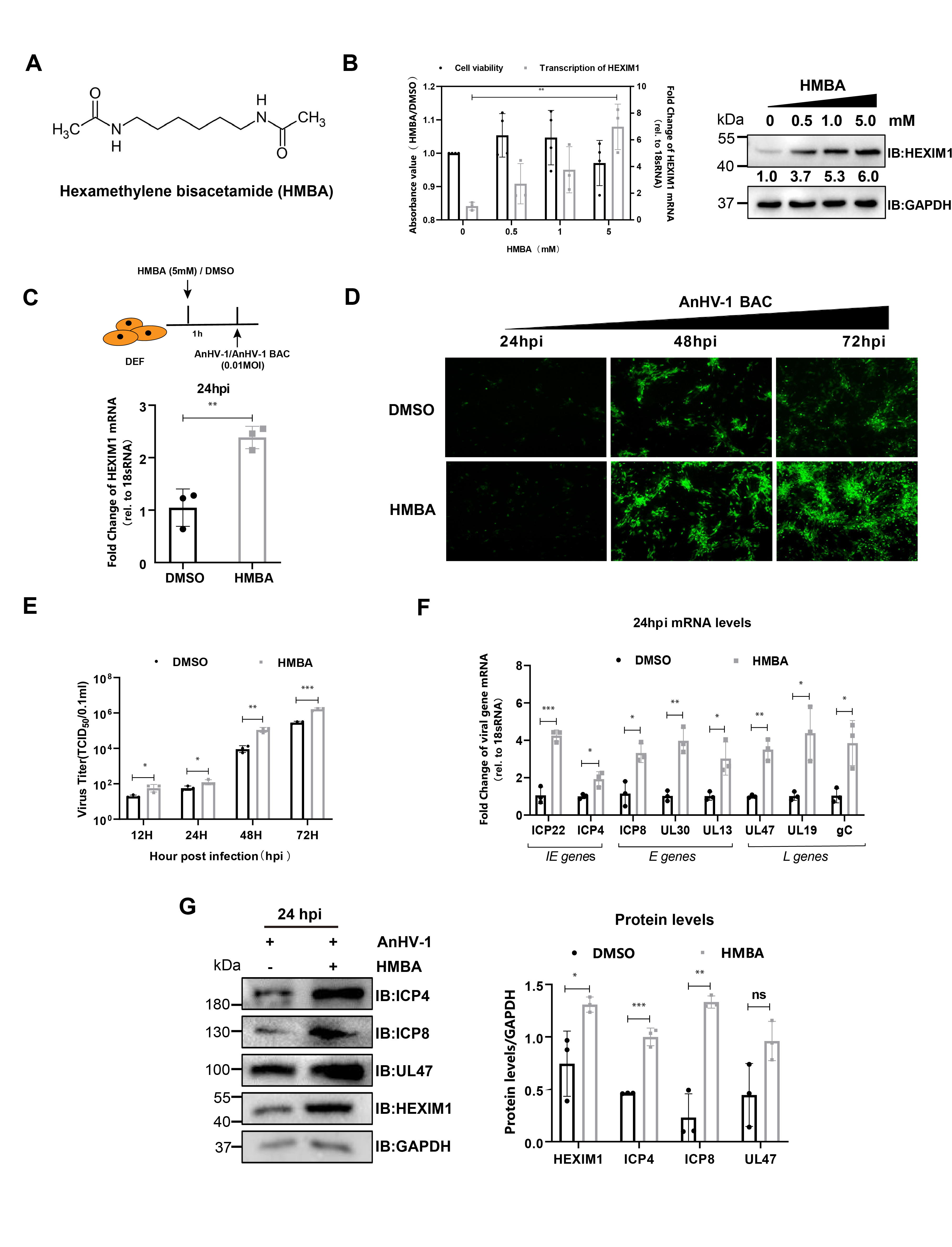

Supplement: Fig. S3 — AnHV-1 replication and viral gene expression are aided by HMBA-induced HEXIM1 expression. [file jvi.01392-23-s0005.tif]

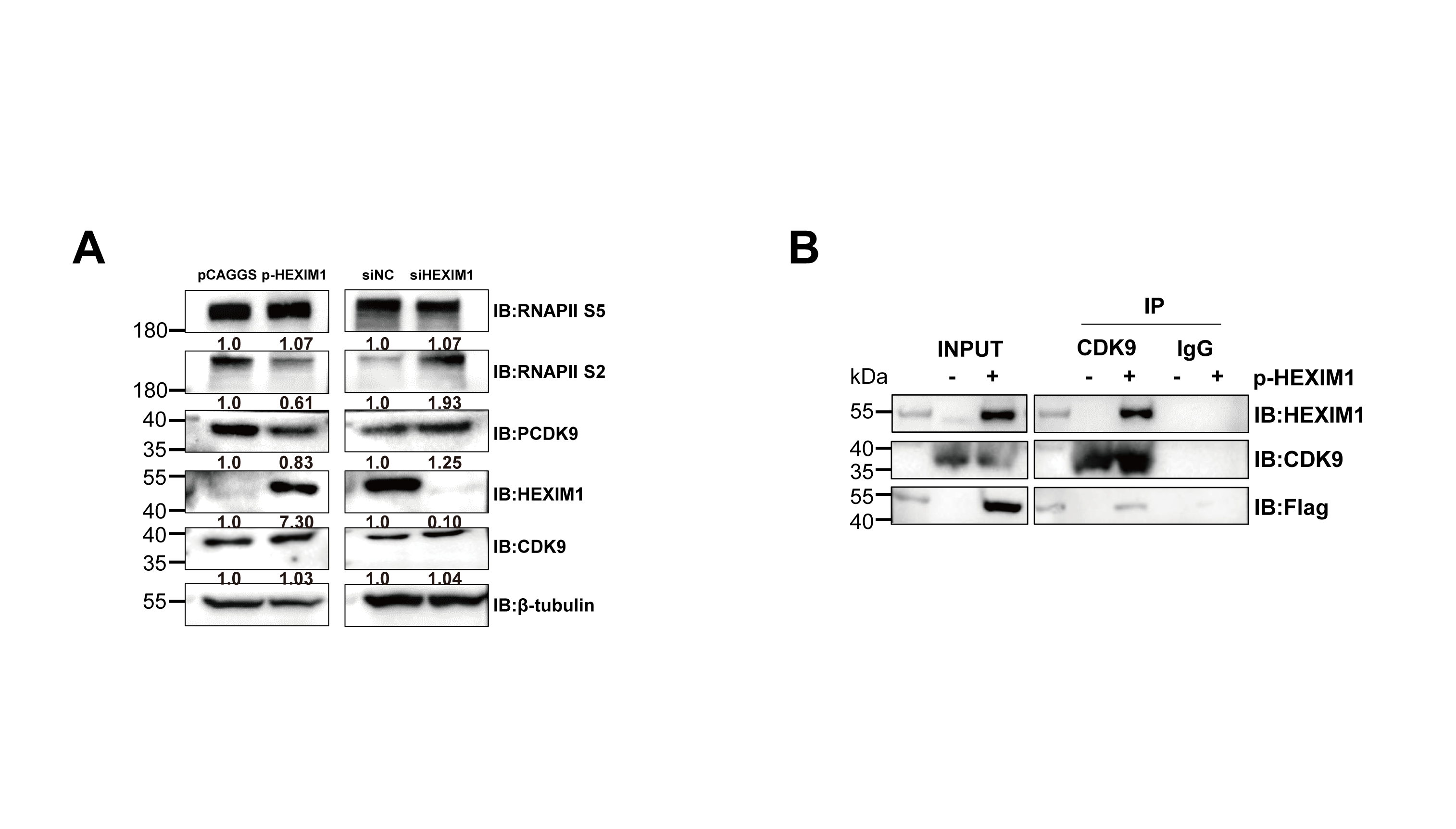

Supplement: Fig. S4 — The effect of HEXIM1 on CDK9 and RNAPII S2 phosphorylation and CDK9-HEXIM1 interaction. [file jvi.01392-23-s0006.tif]

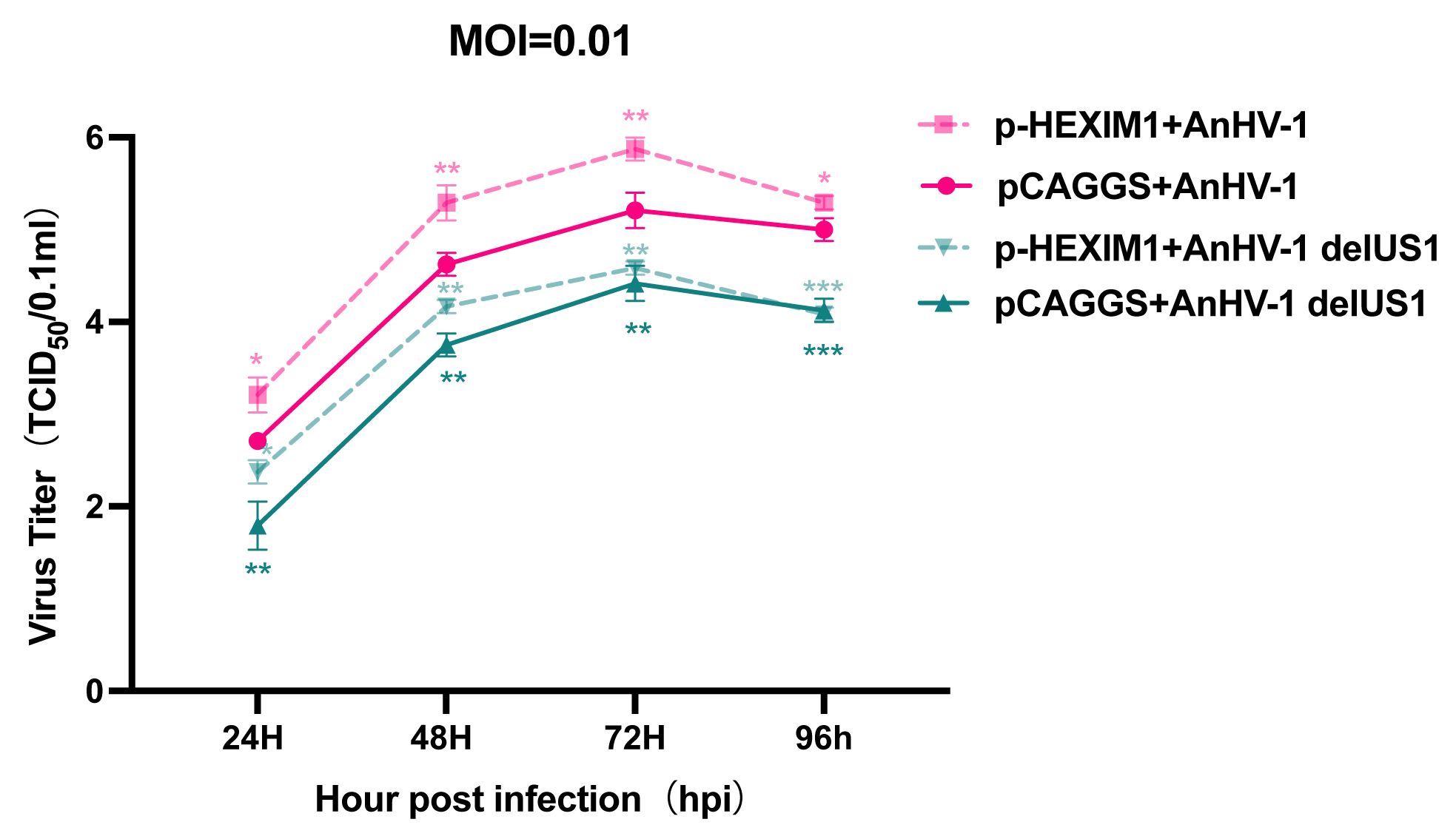

Supplement: Fig. S5 — Influence of HEXIM1 on the proliferation of WT and US1-mutant. [file jvi.01392-23-s0007.tif]
